# Correction: Randomised controlled trial of gradual antipsychotic reduction and discontinuation in people with schizophrenia and related disorders: the RADAR trial (Research into Antipsychotic Discontinuation and Reduction)

**DOI:** 10.1136/bmjopen-2019-030912corr1

**Published:** 2020-07-28

Moncrieff J, Lewis G, Freemantle N, *et al*. Randomised controlled trial of gradual antipsychotic reduction and discontinuation in people with schizophrenia and related disorders: the RADAR trial (Research into Antipsychotic Discontinuation and Reduction). *BMJ Open* 2019;9:e030912.

The authors want to alert readers to the following correction made to the published version.

The co-author name Louise Marston with the affiliation Research Department of Primary Care and Population Health, University College London, London, UK has been missed in the published article.

