# Supplementary material for: Randomised controlled trial of gradual antipsychotic reduction and discontinuation in people with schizophrenia and related disorders: the RADAR trial (Research into Antipsychotic Discontinuation and Reduction)
Source: BMJ Open. 2019 Nov 27;9(11):e030912. doi: 10.1136/bmjopen-2019-030912 (PMC6887002; doi:10.1136/bmjopen-2019-030912)
Supplement: Author's manuscript [file bmjopen-2019-030912.draft_revisions.pdf]

BMJ Open is committed to open peer review. As part of this commitment we make the peer review history of every article we publish publicly available.

When an article is published we post the peer reviewers' comments and the authors' responses online. We also post the versions of the paper that were used during peer review. These are the versions that the peer review comments apply to.

The versions of the paper that follow are the versions that were submitted during the peer review process. They are not the versions of record or the final published versions. They should not be cited or distributed as the published version of this manuscript.

BMJ Open is an open access journal and the full, final, typeset and author-corrected version of record of the manuscript is available on our site with no access controls, subscription charges or pay-per-view fees (http://bmjopen.bmj.com).

If you have any questions on BMJ Open's open peer review process please email info.bmjopen@bmj.com

BMJ Open

A randomised controlled trial of gradual antipsychotic reduction and discontinuation in people with schizophrenia and related disorders: The RADAR trial (Research into Antipsychotic Discontinuation and Reduction): Protocol paper

Journal:	*BMJ Open*
Manuscript ID	bmjopen-2019-030912
Article Type:	Protocol
Date Submitted by the Author:	08-Apr-2019

Complete List of Authors:	Moncrieff, Joanna; University College London and North East London mental health trust, Mental Health Sciences<br>Lewis, Glyn; UCL Psychiatric Epidemiology, Mental Health Sciences Unit<br>Freemantle, Nick; University College London, Comprehensive Clinical Trials Unit<br>Johnson, Sonia; UCL, Division of Psychiatry<br>Barnes, Thomas; Imperial College London, Centre for Mental Health<br>Morant, Nicola; University College London,<br>Pinfold, Vanessa; McPin Foundation,<br>Hunter, Rachael; University College London, Research Dept of Primary Care and Population Health<br>Kent, Lyn<br>Smith, Ruth<br>Darton, Katherine<br>Horne, Robert; University College London, School of Pharmacy<br>Crellin, Nadia; UCL, Division of Psychiatry<br>Cooper, Ruth; Queen Mary University of London, Unit for Social and Community Psychiatry<br>Priebe, Stefan; Barts and the London School of Medicine and Dentistry, University of London, Unit of Social and Community Psychiatry

Keywords:	antipsychotics, schizophrenia, psychosis, antipsychotic maintenance, schizophrenia

SCHOLARONE™
Manuscripts

A randomised controlled trial of gradual antipsychotic reduction and discontinuation in people with schizophrenia and related disorders: The RADAR trial (Research into Antipsychotic Discontinuation and Reduction)

Protocol paper

Joanna Moncrieff[1,2], Glyn Lewis[1], Nicholas Freemantle[1], Sonia Johnson[1], Thomas R. E. Barnes[3], Nicola Morant[1], Vanessa Pinfold[4], Rachael Hunter[5], Lyn Kent, Ruth Counter-Smith, Katherine Darton, Rob Horne[6], Nadia Crellin[1,2], Ruth Cooper[7], Stefan Priebe[7].

[1]Division of Psychiatry, University College London, London, UK

[2]Research & Development Department, Goodmayes Hospital, North East London NHS Foundation Trust, Essex, UK

[3]Centre for Psychiatry, Imperial College London, London, UK

[4]McPin Foundation, London, UK

[5]Research Department of Primary Care and Population Health, Royal Free Medical School, London, UK

[6]School of Pharmacy, University College London, London, UK

[7]Newham Centre for Mental Health, Unit for Social and Community Psychiatry, Queen Mary University of London, London, UK

Correspondence to:

Dr Joanna Moncrieff, Division of Psychiatry, Maple House, London, W1T 7NF j.moncrieff@ucl.ac.uk

Word count: 3650 (not including references, abstract or acknowledgements, author contributions funding and conflict of interest statement).

For peer review only - http://bmjopen.bmj.com/site/about/guidelines.xhtml

ABSTRACT

Introduction

Although antipsychotic medication is effective in reducing the acute symptoms of psychosis, such treatment has a range of potentially serious and debilitating adverse effects and is often disliked by patients. It is therefore essential that it is only used when necessary and when the benefits outweigh the harms. Although multiple trials conducted with people with schizophrenia indicate an increased risk of relapse in the short-term following abrupt antipsychotic discontinuation, there is little evidence about the long-term outcome of a gradual process of reduction and discontinuation of antipsychotic medication on social functioning, relapse and other outcomes.

Methods and analysis

This is a multi-centre, randomised controlled trial involving people with schizophrenia and related disorders who have had more than one episode. Participants are randomised to have a clinically-supervised, gradual reduction of their antipsychotic medication, leading to discontinuation if possible, or to continue with maintenance treatment. Blinded follow-up assessments are conducted at 6, 12 and 24 months and the primary outcome is social functioning, as measured by the Social Functioning Scale, at 24 months. A minimum of 134 evaluable participants will provide 90% power to detect a five-point difference. Secondary outcomes include severe relapse (admission to hospital) and all relapses, as identified by an independent and blinded endpoint committee. Other secondary outcomes include symptoms as measured by the Positive and Negative Syndrome Scale (PANSS), quality of life, adverse effects, self-rated recovery and neuropsychological measures. Enrolment started in 2016.

Ethics and dissemination

Ethical approval was initially obtained on 27/10/2016 (United Kingdom Research Ethics Committee reference 16/LO/1507). Results will be published in peer–reviewed journals and disseminated to the public and the media.

Trial registration ISRCTN 90298520. Pre-results.

For peer review only - http://bmjopen.bmj.com/site/about/guidelines.xhtml

Strengths and limitations

- The trial will be one of the first to provide data on the outcome of a gradual process of antipsychotic reduction and discontinuation in people with schizophrenia and related disorders
- The trial will provide relatively long-term outcome data on social functioning as well as relapse and other outcomes
- There are likely to be some deviations from the planned treatment strategies
- Longer-term follow-up would be desirable and will be initiated in the future

For peer review only

For peer review only - http://bmjopen.bmj.com/site/about/guidelines.xhtml

A randomised controlled trial of gradual antipsychotic reduction and discontinuation in people with schizophrenia and related disorders: The RADAR trial (Research into Antipsychotic Discontinuation and Reduction)

Protocol paper

INTRODUCTION

Schizophrenia and related conditions affect up to 1% of the population (1), and are associated with long-term suffering and disability, premature death, physical illness and high costs to individuals and society (2). Recommended treatment for people with recurrent episodes consists of continuing antipsychotic medication,(3,4). Current guidelines do not recommend attempts at reduction or discontinuation of antipsychotics after the first episode, and in practice, antipsychotic treatment is often 'for life'. In spite of ongoing treatment, many people remain functionally impaired. In one study 25% of people with schizophrenia had severe social disabilities after 15 years, and only 14% had none (5).

The evidence base for long-term antipsychotic treatment consists of studies showing lower relapse rates with maintenance treatment compared with discontinuation (6). However, there are acknowledged methodological problems with these studies (7). In addition, several long-term non-randomised cohorts studies find worse outcomes in people who take continuous treatment compared to those who do not, although confounding by indication is likely to be relevant (8,9). Criticism of randomised controlled trials includes the fact that most focus on relapse and neglect other outcomes. Follow-up is generally short; only six of 65 studies included in a recent meta-analysis by Leucht et al (6) had followed the participants up for more than a year, and longer duration of follow-up was found to be associated with less difference between maintenance and discontinuation (6,10). Moreover, relapse rates may have been inflated by abrupt discontinuation and misidentification of withdrawal-related adverse effects (11,12). Some evidence suggests gradual discontinuation may reduce risk of relapse compared with abrupt discontinuation (13), although this difference was not confirmed in the analysis by Leucht et al (6). However, the average taper of 28 days for gradual medication withdrawal may not have prevented discontinuation-related effects in those people who had been taking antipsychotics for many years.

Long-term antipsychotic medication is associated with potentially serious physical complications, including diabetes, tardive dyskinesia, and cardiovascular disease (14–17).

For peer review only - http://bmjopen.bmj.com/site/about/guidelines.xhtml

Other adverse effects, such as sexual dysfunction, sedation, emotional blunting and akathisia may be debilitating and unpleasant (18–20). Previous research has found that many patients find the adverse effects of antipsychotics burdensome, and would like to try and discontinue the treatment at some stage(21–23).

There are few long-term follow-ups of people from randomised studies of antipsychotic discontinuation. A 7-year follow-up of an 18 month open trial conducted with people with first episode psychosis found that assignment to a gradual antipsychotic reduction programme was associated with better rates of social recovery and equal rates of relapse compared to maintenance treatment (24). Improved levels of neurocognitive performance exhibited by those randomised to antipsychotic reduction at 18 months suggest a possible mechanism for the long-term differences in functional outcome (25). In contrast, recent data from a 10-year follow-up of a one-year, placebo-controlled trial of quetiapine in people with first-episode psychosis reported higher rates of a composite 'poor outcome' in people originally randomised to placebo. There were no differences, however, in overall symptom measures, social functioning or quality of life (26).

Therefore, although continuing antipsychotic treatment has become the norm, it remains unclear whether it has an optimal risk-benefit balance for all people with psychosis or schizophrenia. In particular, more evidence on the effects of gradual reduction of antipsychotics on short and longer term outcomes could inform practice in this area. Further studies are currently being conducted in the first-episode psychosis population (27). To date, however, there is no study using gradual and flexible reduction with long-term follow-up in people with more than one episode.

For peer review only

Aims of trial

The current trial was designed to compare the benefits and harms of a gradual programme of dose reduction and discontinuation of antipsychotic treatment, under clinician guidance, with maintenance treatment in people with a diagnosis of schizophrenia or a related disorder. In particular, the study was set up to test whether such a strategy can improve functional outcomes in people with recurrent or chronic psychosis while minimising the risk of worsening symptoms or relapse.

For peer review only - http://bmjopen.bmj.com/site/about/guidelines.xhtml

METHODS AND ANALYSIS

Study design

The trial consists of an open, parallel group randomised trial with individual randomisation. Participants and clinicians are aware of allocation, but outcome assessors are maintained blind to intervention arm as far as possible. The trial lasts for two years, with follow-up assessments conducted at 6, 12 and 24 months post randomisation. The trial design is presented in Figure 1. Ethical approval includes the possibility of conducting a longer-term follow-up, and resources will be sought to support this at a later date.

Interventions

The antipsychotic reduction protocol was developed after consultation with professionals, academics and the trial's RADAR Lived Experience Advisory Panel (LEAP). Both the antipsychotic reduction and maintenance protocols are administered by treating psychiatrists. For those participants randomised to the antipsychotic medication reduction arm, an individualised reduction schedule is devised by the research team, based on the participant's initial antipsychotic regimen. The dose is reduced incrementally, and the treating psychiatrists are asked to see the participants who have been randomised to antipsychotic reduction approximately every two months for the duration of the reduction, to adjust the medication regimen and monitor mental state. The participants are offered the option to discontinue antipsychotic medication completely if the reduction progresses well, or to reduce to a 'minimum' dose, defined as the equivalent of 2mg of haloperidol a day or less. This is twice the dose that was defined as a 'low dose' in the Dutch first episode study (24), since some research suggests that doses of antipsychotic medication in people with first episodes should be around half those recommended for people with more established disorders (28,29). Guidance on the antipsychotic reduction strategy stresses the need for flexibility, and includes a suggested protocol for the treatment of adverse reactions to withdrawal or symptom exacerbation.

Participants randomised to maintenance treatment are requested not to make major reductions in their dose of antipsychotic medication during the trial period. Increases in dose are permitted within the protocol, as are changes to a different antipsychotic agent at the same equivalent dose and minor dose reductions to address side effects.

For peer review only - http://bmjopen.bmj.com/site/about/guidelines.xhtml

Participants are monitored by their care team according to usual practice. Patient records are scrutinised by members of the research team every two months to monitor the progress of the antipsychotic reduction and adherence to the maintenance protocol. Any deviations, such as participants allocated to reduction not starting or halting their reduction, are discussed with the treating clinician.

Inclusion and exclusion criteria

The eligibility criteria are detailed in Table 1. They are designed to make the trial as generalizable as possible to routine clinical practice, whilst minimising the risks of antipsychotic reduction in those with a history of posing a serious risk to self or others.

Table 1. Eligibility criteria

**Inclusion criteria**	
1.	Aged 18 years or older
2.	A clinical and/or ICD10 diagnosis of schizophrenia, schizoaffective disorder, delusional disorder or other non-affective psychosis
3.	More than one previous episode or psychotic exacerbation, or a single episode lasting more than one year
4.	Prescribed continuing antipsychotic medication
**Exclusion criteria**	
1.	Lack of capacity to consent to the trial.
2.	Insufficient command of spoken English to understand trial procedures
3.	Subject to a section of the Mental Health Act (MHA) that includes a requirement to take antipsychotic medication
4.	Clinician considers there will be a serious risk of harm to self or others
5.	Admitted to hospital or treated by a Home Treatment or Crisis Team within the last month
6.	Women who have a confirmed pregnancy
7.	Women who are breast-feeding

For peer review only - http://bmjopen.bmj.com/site/about/guidelines.xhtml

8. Involvement in another Investigational Medicinal Product (IMP) trial

---

Outcome measures

The primary outcome is social functioning at two-year follow-up. This will be measured using the Social Functioning Scale (SFS). The scale was developed in 1990, and has good reliability (30,31). It can demonstrate change over time (32–34) and distinguishes different types of antipsychotic treatment (32–35). It also distinguishes between people in remission and those with an ongoing episode (36), people with long versus short DUP (37), people who convert to psychosis versus those who do not in an 'ultra-high risk' cohort (33), and between people who are employed versus unemployed (30). This scale was preferred to the Groningen Social Disability Scale (GSDS) (5), which was used in the Dutch first-episode study, because of the GSDS is long, requires lengthy training and has a strong focus on relationships rather than functioning.

The main measure of the safety of the trial is severe relapse which is defined as admission to acute psychiatric in-patient treatment. This was selected because it is an objective criterion, can be precisely dated and can be obtained through patient records. Previous antipsychotic withdrawal studies have defined relapse in numerous different ways, with no consensus on defining criteria. Therefore, the current trial will also use an expert endpoint committee to assess the presence or absence of relapse based on blinded information from clinical case-notes, based on pre-defined criteria and guidance.

Other outcome measures include: symptoms as measured by the Positive and Negative Syndrome Scale (PANSS) (38), subjective quality of life as measured by the Manchester Short Assessment of quality of life (MANSA) (39), adverse effects of antipsychotics measured by a modified version of the Glasgow Antipsychotic Side-effect Scale (GASS) (40), body weight, sexual dysfunction as measured by the Arizona Sexual Experiences Scale (ASEX) (41), Work Productivity and Activity Impairment- General Health (WPAI) questionnaire (42), neuropsychological function (measured by a brief battery of tests designed for this trial, recovery as measured by the Questionnaire about the Process of Recovery (QPR) (43), ICECAP-A (quality of life), and EQ-5D-5L (quality of life).

In addition, process data will be collected, including dose of antipsychotic medication (in haloperidol equivalents), patient satisfaction as measured by the Client Satisfaction

For peer review only - http://bmjopen.bmj.com/site/about/guidelines.xhtml

Questionnaire CSQ (44) and antipsychotic medication adherence as measured by Medication Adherence Rating Scale MARS-5 (45). Table 2 shows the time points at which each measure will be completed.

Table 2. Time points at which different outcomes will be assessed

*A = Demographic information (selected sections including weight and use of illicit drugs and alcohol); B = Diagnosis (established from clinical records); C = Social Functioning Scale (SFS); D = Positive and Negative Syndrome Scale (PANSS); E = Glasgow Antipsychotic Side-effect Scale (GASS); F = Client Satisfaction Questionnaire (CSQ-8); G = Manchester Short Assessment of quality of life (MANSA); H = Neuropsychological function tests; I = Medication Adherence Rating Scale (MARS-5); J = Relapse questionnaire ; K = Serious Adverse Events; L = EQ-5D-5L; M = ICECAP-A; N = Client Service Receipt Inventory ; O = Work Productivity and Activity Questionnaire; P = Schedule for economic data from patient records; Q = Questionnaire about the Process of Recovery (QPR); R = Arizona Sexual Experiences Scale (ASEX).

	**Baseline**	**Follow-up**				
**Visit Number**	**1**	**2 Pilot trial 3-month data collection**	**3 6-month follow-up**	**4 12-month follow-up**	**5 24-month follow-up**	**6 Qualitative evaluation**
**Informed Consent**	X					X
**Eligibility determination**	X					
**Protocol Assessments**	A-I, L-O, Q, R	K,	C-G, I-O, Q, R	C-O,Q, R	C- R	Indicative topic guide with sample of participants and psychiatrists
**Randomisation**	X					
**IMP administration**	X	X	X	X	X	
**Adverse Events review**	X	X	X	X	X	
**Medical notes review for prescribing information and fidelity to intervention protocols**	X	X	X	X	X	
**Concomitant Medication review**	X	X	X	X	X	

For peer review only - http://bmjopen.bmj.com/site/about/guidelines.xhtml

Risk reduction

The eligibility criteria are designed to exclude people with known high risks of causing harm to themselves or other people. In addition, the gradual nature of the antipsychotic reduction will enable detection and treatment of early signs of relapse. All participants will receive usual care and monitoring of their mental state and behaviour by their clinical team. Those randomised to antipsychotic reduction will have increased contact with a psychiatrist for the duration of the reduction, mirroring usual clinical practice with someone undergoing a significant reduction of medication.

Qualitative assessment

There will be a qualitative sub-study involving around 20-30 participants from the antipsychotic reduction group and around 12 clinicians with experience of delivering the antipsychotic reduction programme. This study has two aims: to collect data on participant and clinician experience of trial processes, and to explore in detail experiences of antipsychotic reduction and discontinuation from the patient perspective. Participants will be identified towards the end of follow up, using purposive sampling to obtain variation in clinical profile, experience of reduction, completion of the antipsychotic reduction protocol and experience of relapse.

Participants will be interviewed using a semi-structured interview schedule developed in collaboration with the RADAR Lived Experience Advisory Panel (LEAP). This will explore patients' experiences of the intervention and its impact on their mental health and wider lives, the acceptability of antipsychotic reduction, satisfaction with available support, and responses to adverse events. Interviews will be conducted with early participants following the 24-month follow-up interviews so as not to confound the main trial results. Two members of the LEAP plan to be involved in conducting interviews, analysis and write up. Interviews with clinicians will explore their experiences of implementing different aspects of the antipsychotic reduction strategy, relations with the research team, acceptability of medication monitoring procedures, and responses to adverse events. The data will be analysed using thematic analysis.

For peer review only - http://bmjopen.bmj.com/site/about/guidelines.xhtml

For peer review only

Statistical analysis

The primary outcome (Social Functioning Scale, SFS) will be analysed using generalised mixed models, accounting for baseline and treatment periods. The principal analysis will be undertaken using the intention to treat population, including all available data, without imputation. Supportive analyses will include analysis of different time periods, analysis using repeated measurements (where all subjects with data at one or more post intervention assessment will be included), exploring potential centre effects and making pessimistic assumptions by group on the patient outcome (to assess the thresholds to the impact of 'missingness') because of the likely pattern of data missing not at random.

The proportions of patients who experience a 'severe relapse' (psychiatric hospital admission) will be compared between the randomised groups at two year follow-up, and survival analysis using Cox constant proportional hazards models including centres as marginal frailty terms will also be performed.

We will undertake supportive threshold analyses where any loss to follow up will be considered according to extreme data patterns – e.g. assuming all those who were lost in the antipsychotic withdrawal group had a relapse and all those in the maintenance group did not, and thus quantifying the extent of possible effects of missingness and thus the robustness of the main result.

A full statistical analysis plan will be produced a priori, describing planned subgroup analyses and with detailed description of the statistical processes to be used.

Economic analysis

The economic evaluation will evaluate the cost-effectiveness of the antipsychotic reduction strategy compared to maintenance treatment over 24 months. The principal analysis will be the incremental cost per quality adjusted life year gained from the health and social services cost perspective using the EQ-5D-5L to calculate quality adjusted life years (QALYs) as recommended by NICE (46). A secondary analysis will include the cost per capability adjusted life year (CALY) gained of the antipsychotic reduction strategy compared to maintenance treatment over 24 months using the ICECAP-A, argued to have better face validity (47).

For peer review only - http://bmjopen.bmj.com/site/about/guidelines.xhtml

Additional sensitivity analyses will explore the cost-effectiveness of antipsychotic reduction compared to maintenance capturing the QoL loss associated with antipsychotics side effects using specific disutility values for antipsychotic side effects from the published literature (48).

The Work Productivity and Activity Impairment- General Health (WPAI) questionnaire and associated formula (42) will be used to give a monetary value to impact on employment. Health and social care data costs using published sources will be used to calculate costs for the primary health and social care analysis. A secondary analysis will be conducted from a societal perspective to capture the impact on employment, criminal justice, benefits, family and close others

All costs and outcomes will be discounted in line with NICE guidance at 3.5% per year. Bootstrapping will be used to construct confidence intervals for total mean costs, QALYs and CALYs and to construct cost effectiveness acceptability curves and cost-effectiveness planes. Missing data will be handled in the same way as stated by the statistical analysis plan.

Sample size calculation

The primary outcome is social functioning, which will be measured by the Social Functioning Scale (30). The literature suggests a minimum difference of between four and five points would be clinically significant, since a difference of four to five points appears to differentiate between patients with good and poor outcomes (36,49,50).

Using a conventional α of 5% (two sided) and taking a SD of 8.8 derived from the literature,(51), a sample size of 134 evaluable participants is required to provide 90% power to identify a five point difference, and a sample of 206 is required to identify a four point difference.

The trial also aimed to be powered to establish the safety of the antipsychotic reduction strategy by powering it to detect whether there is a difference in rates of 'severe' relapse (psychiatric hospitalisation), the principal safety measure, between the intervention arms. We believe that an increased risk of up to 10% would be acceptable to many clinicians and patients if it is balanced by the important outcomes of an improvement in social functioning and reduced side

For peer review only - http://bmjopen.bmj.com/site/about/guidelines.xhtml

For peer review only

effects. We derived event rates for hospitalisation from those reported in the Leucht et al (6) meta-analysis of antipsychotic discontinuation studies.

We conducted a non-inferiority calculation using a 10% margin of difference. Using a non-inferiority boundary of 10% event rates (severe relapse), with an alpha of 0.05, requires a sample size of 372 for 90% power to exclude a difference of 10% between groups. Adding 15% for attrition brings this up to 402. The lower confidence interval on the absolute scale would exclude a difference of 10% in the situation where non inferiority was achieved. 402 participants provides 90% power to detect a difference of 3.1 points on the primary efficacy outcome (the SFS), including an allowance of 15% for attrition or unexpected challenges to the assumptions.

It is acknowledged that the non-inferiority approach may be unhelpful in these circumstances, as different people may have substantially different views on what is a reasonable non-inferiority boundary. Further, the assumptions required on issues such as the event rate for the non-inferiority assumption are strong, and modest changes in rates can have a substantial impact on the statistical power of the study making the proposed non inferiority boundary unhelpful in certain circumstances. Thus the principal aim of the study with regards to severe relapse is to provide a robust estimate of the event rates in each randomised condition, and the difference between them, describing measurement error with appropriate confidence intervals in order to allow individual patients and their clinicians to make an informed decision on treatment options.

PATIENT AND PUBLIC INVOLVEMENT

Testing this research question is a response to a long-standing demand from the service user and carer community for an alternative to long-term antipsychotic medication. Many service users and carers are extremely concerned about the possible long-term side effects of antipsychotic medication. The study has four co-applicants working with a PPI perspective attending project management meetings; a carer, a service user, a public member and a staff member of a relevant charity. There is also a LEAP (Lived Experience Advisory Panel), made up of people with expertise in antipsychotic medication through personal use or as a carer for someone with psychosis. It meets regularly to discuss the progress of the study and to contribute to its development, for example, advising on the antipsychotic reduction strategy. The LEAP will be particularly involved in the qualitative study as part of the process

For peer review only - http://bmjopen.bmj.com/site/about/guidelines.xhtml

evaluation. They will also be involved in providing feedback on study outputs including a regular newsletter and disseminating research results. Results of the study will be disseminated to participants at their request.

ETHICS AND DISSEMINATION

The trial received approval from the Brent Ethics committee on 27/10/2016. The Research Ethics Committee (REC) reference is 16/LO/1507. The trial was registered with the International Standard Randomised Controlled Trials register on 7/2/2017, registration number: ISRCTN90298520 and with ClinicalTrials.gov on 15/6/2018.

Results will be published in peer-reviewed journals and disseminated to the public and the media.

Current trial status

Recruitment started in March 2016 and will continue until 2020.

DISCUSSION

Better evidence is required about the balance of risks and benefits of a supported programme of antipsychotic reduction and discontinuation under the guidance of a clinician for the many people who have a diagnosis of schizophrenia or recurrent psychoses. The current study will be the first study conducted with people with more than one psychotic episode to employ a gradual and supported method of antipsychotic reduction, to look at a wide range of outcomes giving priority to social functioning, and to follow people up for a reasonable duration. Further follow-ups are envisaged after the official end of the trial in order to provide data on longer-term outcomes.

The Dutch first-episode study suggested that long-term outcomes after a period of antipsychotic dose reduction differ from short-term outcomes (24,52). Although relapse rates are increased initially compared with maintenance treatment, over time these equalise. Social functioning, which was not affected in the short-term, was considerably improved by the time of the seven-year follow-up assessment. Social functioning is a measure of independence and personal efficacy. If the current trial results in improvements in social functioning this could

For peer review only - http://bmjopen.bmj.com/site/about/guidelines.xhtml

reduce an individual's reliance on services and produce significant economic benefits. From the individual's point of view, better social functioning reflects a more fulfilling and socially integrated life. A strategy that can successfully reduce the use of antipsychotic medication is also likely to be associated with health benefits and improvements in quality of life secondary to the reduction of adverse effects.

The outcomes of the current trial will provide good evidence to inform patients and clinicians about the likely consequences of reducing and discontinuing long-term antipsychotic treatment in a gradual manner in a clinical setting. Many patients currently want to consider this option, but existing data on the range of relevant outcomes is limited. Providing further information will facilitate a more collaborative approach to long-term antipsychotic treatment.

For peer review only

For peer review only - http://bmjopen.bmj.com/site/about/guidelines.xhtml

PATIENT CONSENT FOR PUBLICATION: Not required

ACKNOWLEDGEMENTS

This research is sponsored by University College London. The RADAR research team acknowledges the support of the National Institute for Health Research Clinical Research Network, Priment Clinical Trials Unit and North East London NHS Foundation Trust. The research team also thank the RADAR Lived Experience Advisory Panel (LEAP) for their expertise, guidance and support. With thanks also to the independent members of the Programme Steering Committee and the Data and Safety Monitoring Board.

AUTHORS' CONTRIBUTIONS

JM developed the original concept for the trial and drafted the protocol. All authors contributed to the trial design and methodology. NC and RC provided trial management and updated the protocol. JM drafted the manuscript, and JM and NC edited subsequent drafts. All authors reviewed and commented on drafts of the paper and approved the final manuscript version.

FUNDING

This report is independent research funded by the National Institute for Health Research (Programme Grants for Applied Research, Research into Antipsychotic Discontinuation And Reduction (RADAR), RP-PG-0514-20004). The views expressed in this publication are those of the author(s) and not necessarily those of the NHS, the National Institute for Health Research or the Department of Health.

COMPETING INTERESTS

TREB: In the last three years, TREB has been a member of scientific advisory boards for Otsuka/Lundbeck, Newron Pharmaceuticals and Gedeon Richter and received speaker fees from Janssen.

GL has acted as an expert witness in cases concerning litigation about antidepressants.

No other authors have competing interests to declare.

For peer review only - http://bmjopen.bmj.com/site/about/guidelines.xhtml

REFERENCES

1. McGrath J, Saha S, Chant D, Welham J. Schizophrenia: A Concise Overview of Incidence, Prevalence, and Mortality. *Epidemiol Rev* 2008;14:30(1):67–76. https://doi.org/10.1093/epirev/mxn001

2. McCrone P, Dhanasiri S, Patel A, Knapp.M., Lawton S. Paying the price. the cost of mental health care in England to 2026. London: Kings Fund; 2008.

3. Hasan A, Falkai P, Wobrock T, Lieberman J, Glenthoj B, Gattaz WF, et al. World Federation of Societies of Biological Psychiatry (WFSBP) Guidelines for Biological Treatment of Schizophrenia, Part 2: Update 2012 on the long-term treatment of schizophrenia and management of antipsychotic-induced side effects. *World J Biol Psychiatry* 2013;14(1):2–44. https://doi.org/10.3109/15622975.2012.739708

4. National Institute for Health and Care Excellence. Psychosis and schizophrenia in adults: treatment and management. London: NICE; 2014.

5. Wiersma D, Wanderling J, Dragomirecka E, Ganev K, Harrison G, an der HW, et al. Social disability in schizophrenia: its development and prediction over 15 years in incidence cohorts in six European centres. *Psychol Med* 2000;30(5):1155-67. https://doi.org/10.1017/S0033291799002627

6. Leucht S, Tardy M, Komossa K, Heres S, Kissling W, Davis JM. Maintenance treatment with antipsychotic drugs for schizophrenia. *Cochrane Database Syst Rev* 2012;5:CD008016. https://doi.org/10.1002/14651858.CD008016.pub2

7. Leucht S, Heres S, Hamann J, Kane JM. Methodological Issues in Current Antipsychotic Drug Trials. *Schizophr Bull* 2008;9;34(2):275–85. https://doi.org/10.1093/schbul/sbm159

8. Harrow M, Jobe TH, Faull RN. Do all schizophrenia patients need antipsychotic treatment continuously throughout their lifetime? A 20-year longitudinal study. *Psychol Med* 2012;42(10):2145–55. https://doi.org/10.1017/S0033291712000220

9. Moilanen JM, Haapea M, Jääskeläinen E, Veijola JM, Isohanni MK, Koponen HJ, et al. Long-term antipsychotic use and its association with outcomes in schizophrenia – the Northern Finland Birth Cohort 1966. *Eur Psychiatry* 2016;36:7–14. https://doi.org/10.1016/j.eurpsy.2016.03.002

10. Gilbert PL, Harris MJ, McAdams LA, Jeste DV. Neuroleptic withdrawal in schizophrenic patients. A review of the literature. *Arch Gen Psychiatry* 1995;52(3):173-88. https://doi.org/10.1001/archpsyc.1995.03950150005001

11. Baldessarini RJ, Viguera AC. Neuroleptic withdrawal in schizophrenic patients. *Arch Gen Psychiatry* 1995;52(3):189-92. https://doi.org/10.1001/archpsyc.1995.03950150021002

12. Whitaker R. Anatomy of an Epidemic. New York: Crown Publishers; 2010.

13. Viguera AC, Baldessarini RJ, Hegarty JD, van Kammen DP, Tohen M. Clinical risk following abrupt and gradual withdrawal of maintenance neuroleptic treatment. *Arch Gen Psychiatry* 1997;54(1):49–55. https://doi.org/10.1001/archpsyc.1997.01830130055011

14. Osborn DPJ, Levy G, Nazareth I, Petersen I, Islam A, King MB. Relative Risk of Cardiovascular and Cancer Mortality in People With Severe Mental Illness From the United

For peer review only - http://bmjopen.bmj.com/site/about/guidelines.xhtml

Kingdom's General Practice Research Database. *Arch Gen Psychiatry* 2007;64(2):242-9. https://doi.org/10.1001/archpsyc.64.2.242

15. Ray WA, Chung CP, Murray KT, Hall K, Stein CM. Atypical antipsychotic drugs and the risk of sudden cardiac death. *N Engl J Med* 2009;15;360(3):225-35. https://doi.org/10.1056/NEJMoa0806994

16. Yood MU, deLorenze G, Quesenberry CP, Oliveria SA, Tsai A-L, Willey VJ, et al. The incidence of diabetes in atypical antipsychotic users differs according to agent-results from a multisite epidemiologic study. *Pharmacoepidemiol Drug Saf* 2009;18(9):791–9. https://doi.org/10.1002/pds.1781

17. Correll CU, Schenk EM. Tardive dyskinesia and new antipsychotics. *Curr Opin Psychiatry* 2008;21(2):151-6. https://doi.org/10.1097/YCO.0b013e3282f53132

18. Moncrieff J, Cohen D, Mason JP. The subjective experience of taking antipsychotic medication: a content analysis of Internet data. *Acta Psychiatr Scand* 2009;120(2):102–11. https://doi.org/10.1111/j.1600-0447.2009.01356.x

19. Barbui C, Nose M, Bindman J, Schene A, Becker T, Mazzi MA, et al. Sex Differences in the Subjective Tolerability of Antipsychotic Drugs. *J Clin Psychopharmacol* 2005;25(6):521–6. https://doi.org/10.1097/01.jcp.0000185423.15891.02

20. Hatano M, Kamei H, Kato A, Takeuchi I, Hanya M, Uno J, et al. Assessment of the Latent Adverse Events of Antipsychotic Treatment Using a Subjective Questionnaire in Japanese Patients with Schizophrenia. *Clin Psychopharmacol Neurosci* 2017;31;15(2):132–7. https://doi.org/10.9758/cpn.2017.15.2.132

21. Williams K, Pinfold V. Side Effects: Mental health service users experiences of the side effects of antipsychotic medication. London: Rethink; 2006.

22. Read J. Psychiatric Drugs: Key issues and service user perspectives. Basingstoke, Hants: Palgrave Macmillan; 2009.

23. Morant N, Azam K, Johnson S, Moncrieff J. The least worst option: user experiences of antipsychotic medication and lack of involvement in medication decisions in a UK community sample. *J Ment Health* 2018;4;27(4):322–8. https://doi.org/10.1080/09638237.2017.1370637

24. Wunderink L, Nieboer RM, Wiersma D, Sytema S, Nienhuis FJ. Recovery in Remitted First-Episode Psychosis at 7 Years of Follow-up of an Early Dose Reduction/Discontinuation or Maintenance Treatment Strategy: Long-term Follow-up of a 2-Year Randomized Clinical Trial. *JAMA Psychiatry* 2013;1;70(9):913. https://doi.org/10.1001/jamapsychiatry.2013.19

25. Faber G, Smid HGOM, Van Gool AR, Wunderink L, Wiersma D, van den Bosch RJ. Neurocognition and recovery in first episode psychosis. *Psychiatry Res* 2011;188(1):1–6. https://doi.org/10.1016/j.psychres.2010.11.010

26. Hui CLM, Honer WG, Lee EHM, Chang WC, Chan SKW, Chen ESM, et al. Long-term effects of discontinuation from antipsychotic maintenance following first-episode schizophrenia and related disorders: a 10 year follow-up of a randomised, double-blind trial. *Lancet Psychiatry* 2018;5(5):432–42. https://doi.org/10.1016/S2215-0366(18)30090-7

27. Stürup AE, Jensen HD, Dolmer S, Birk M, Albert N, Nielsen M, et al. TAILOR – tapered discontinuation versus maintenance therapy of antipsychotic medication in patients with newly

For peer review only - http://bmjopen.bmj.com/site/about/guidelines.xhtml

diagnosed schizophrenia or persistent delusional disorder in remission of psychotic symptoms: study protocol for a randomized clinical trial. *Trials* [Internet]. 2017 Dec [cited 2019 Feb 18];18(1). Available from: http://trialsjournal.biomedcentral.com/articles/10.1186/s13063-017-2172-4

28. McEvoy JP, Hogarty GE, Steingard S. Optimal dose of neuroleptic in acute schizophrenia. A controlled study of the neuroleptic threshold and higher haloperidol dose. *Arch Gen Psychiatry* 1991;48(8):739–45. https://doi.org/10.1001/archpsyc.1991.01810320063009

29. Oosthuizen P, Emsley RA, Turner J, Keyter N. Determining the optimal dose of haloperidol in first-episode psychosis. *J Psychopharmacol (Oxf)* 2001;15(4):251–5. https://doi.org/10.1177/026988110101500403

30. Birchwood M, Smith J, Cochrane R, Wetton S, Copestake S. The Social Functioning Scale. The Development and Validation of a New Scale of Social Adjustment for use in Family Intervention Programmes with Schizophrenic Patients. *Br J Psychiatry* 1990;157(06):853–9. https://doi.org/10.1192/bjp.157.6.853

31. Vázquez Morejón AJ, Jiménez Ga-Bóveda R. Social Functioning Scale: new contributions concerning its psychometric characteristics in a Spanish adaptation. *Psychiatry Res* 2000;93(3):247–56. https://doi.org/10.1016/S0165-1781(99)00123-7

32. Ciudad A, Olivares JM, Bousoño M, Gómez JC, Álvarez E. Improvement in social functioning in outpatients with schizophrenia with prominent negative symptoms treated with olanzapine or risperidone in a 1 year randomized, open-label trial. *Prog Neuropsychopharmacol Biol Psychiatry* 2006;30(8):1515–22. https://doi.org/10.1016/j.pnpbp.2006.05.010

33. Jang JH, Shin NY, Shim G, Park HY, Kim E, Jang G-E, et al. Longitudinal Patterns of Social Functioning and Conversion to Psychosis in Subjects at Ultra-High Risk. *Aust N Z J Psychiatry* 2011;45(9):763–70. https://doi.org/10.3109/00048674.2011.595684

34. Sheridan AJ, Drennan J, Coughlan B, O'Keeffe D, Frazer K, Kemple M, et al. Improving social functioning and reducing social isolation and loneliness among people with enduring mental illness: Report of a randomised controlled trial of supported socialisation. *Int J Soc Psychiatry* 2015;61(3):241–50. https://doi.org/10.1177/0020764014540150

35. Koshikawa Y, Takekita Y, Kato M, Sakai S, Onohara A, Sunada N, et al. The Comparative Effects of Risperidone Long-Acting Injection and Paliperidone Palmitate on Social Functioning in Schizophrenia: A 6-Month, Open-Label, Randomized Controlled Pilot Trial. *Neuropsychobiology* 2016;73(1):35–42. https://doi.org/10.1159/000442209

36. Jaracz K, Górna K, Kiejda J, Grabowska-Fudala B, Jaracz J, Suwalska A, et al. Psychosocial functioning in relation to symptomatic remission: A longitudinal study of first episode schizophrenia. *Eur Psychiatry* 2015;30(8):907–13. https://doi.org/10.1016/j.eurpsy.2015.08.001

37. Barnes TRE, Leeson VC, Mutsatsa SH, Watt HC, Hutton SB, Joyce EM. Duration of untreated psychosis and social function: 1-year follow-up study of first-episode schizophrenia. *Br J Psychiatry* 2008;193(03):203–9. https://doi.org/10.1192/bjp.bp.108.049718

38. Kay SR, Fiszbein A, Opler LA. The Positive and Negative Syndrome Scale (PANSS) for Schizophrenia. *Schizophr Bull* 1987;1;13(2):261–76. https://doi.org/10.1093/schbul/13.2.261

For peer review only - http://bmjopen.bmj.com/site/about/guidelines.xhtml

39. Priebe S, Huxley P, Knight S, Evans S. Application and Results of the Manchester Short Assessment of Quality of Life (Mansa). *Int J Soc Psychiatry* 1999;45(1):7–12. https://doi.org/10.1177/002076409904500102

40. Waddell L, Taylor M. A new self-rating scale for detecting atypical or second-generation antipsychotic side effects. *J Psychopharmacol (Oxf)* 2008;22(3):238–43. https://doi.org/10.1177/0269881107087976

41. McGahuey CA, Gelenberg AJ, Laukes CA, Moreno FA, Delgado PL, McKnight KM, et al. The Arizona Sexual Experience Scale (ASEX): reliability and validity. *J Sex Marital Ther* 2000 Jan;26(1):25-40. https://doi.org/10.1080/009262300278623

42. Reilly MC, Zbrozek AS, Dukes EM. The validity and reproducibility of a work productivity and activity impairment instrument. *Pharmacoeconomics* 1993;4(5):353–65. https://doi.org/10.2165/00019053-199304050-00006

43. Law H, Neil ST, Dunn G, Morrison AP. Psychometric properties of the Questionnaire about the Process of Recovery (QPR). *Schizophr Res* 2014;156(2–3):184–9. https://doi.org/10.1016/j.schres.2014.04.011

44. Atkisson CC, Zwick R. The Client Satisfaction Questionnaire: Psychometric properties and correlations with service utilisation and psychotherapy outcome. *Evaluation and Program Planning* 1982;5(3):233-7. https://doi.org/10.1016/0149-7189(82)90074-X

45. Mahler C, Hermann K, Horne R, Ludt S, Haefeli WE, Szecsenyi J, et al. Assessing reported adherence to pharmacological treatment recommendations. Translation and evaluation of the Medication Adherence Report Scale (MARS) in Germany. *J Eval Clin Pract* 2010;16(3):574-9. https://doi.org/10.1111/j.1365-2753.2009.01169.x

46. EuroQol Group. EuroQol--a new facility for the measurement of health-related quality of life. *Health Policy* 1990;16(3):199-208. https://doi.org/10.1016/0168-8510(90)90421-9

47. Brazier J, Connell J, Papaioannou D, Mukuria C, Mulhern B, Peasgood T, et al. A systematic review, psychometric analysis and qualitative assessment of generic preference-based measures of health in mental health populations and the estimation of mapping functions from widely used specific measures. *Health Technol Assess* 2014;18(34):vii-xxv,1. https://doi.org/10.3310/hta18340

48. Briggs A, Wild D, Lees M, Reaney M, Dursun S, Parry D, et al. Impact of schizophrenia and schizophrenia treatment-related adverse events on quality of life: direct utility elicitation. *Health Qual Life Outcomes* 2008;6(1):105. https://doi.org/10.1186/1477-7525-6-105

49. Hellvin T, Sundet K, Vaskinn A, Simonsen C, Ueland T, Andreassen OA, et al. Validation of the Norwegian version of the Social Functioning Scale (SFS) for schizophrenia and bipolar disorder: Validation of the SFS (Norwegian version). *Scand J Psychol* 2010;51(6):525–33. https://doi.org/10.1111/j.1467-9450.2010.00839.x

50. Leeson VC, Harrison I, Ron MA, Barnes TRE, Joyce EM. The Effect of Cannabis Use and Cognitive Reserve on Age at Onset and Psychosis Outcomes in First-Episode Schizophrenia. *Schizophr Bull* 2012;1;38(4):873–80. https://doi.org/10.1093/schbul/sbq153

For peer review only

For peer review only - http://bmjopen.bmj.com/site/about/guidelines.xhtml

51. Slupczynska-Kossobudzka E, Boguszewska L. Effects of Community Mobile Team Intervention in the Drewnica Hospital Catchment Area. 1. Patient Outcome. *Int J Soc Psychiatry* 1999;45(3):207–15. https://doi.org/10.1177/002076409904500308

52. Wunderink L, Nienhuis FJ, Sytema S, Slooff CJ, Knegtering R, Wiersma D. Guided discontinuation versus maintenance treatment in remitted first-episode psychosis: relapse rates and functional outcome. *J Clin Psychiatry* 2007;68(5):654-61. https://doi.org/10.4088/JCP.v68n0502

For peer review only

For peer review only - http://bmjopen.bmj.com/site/about/guidelines.xhtml

Figure 1. Trial Flow Chart

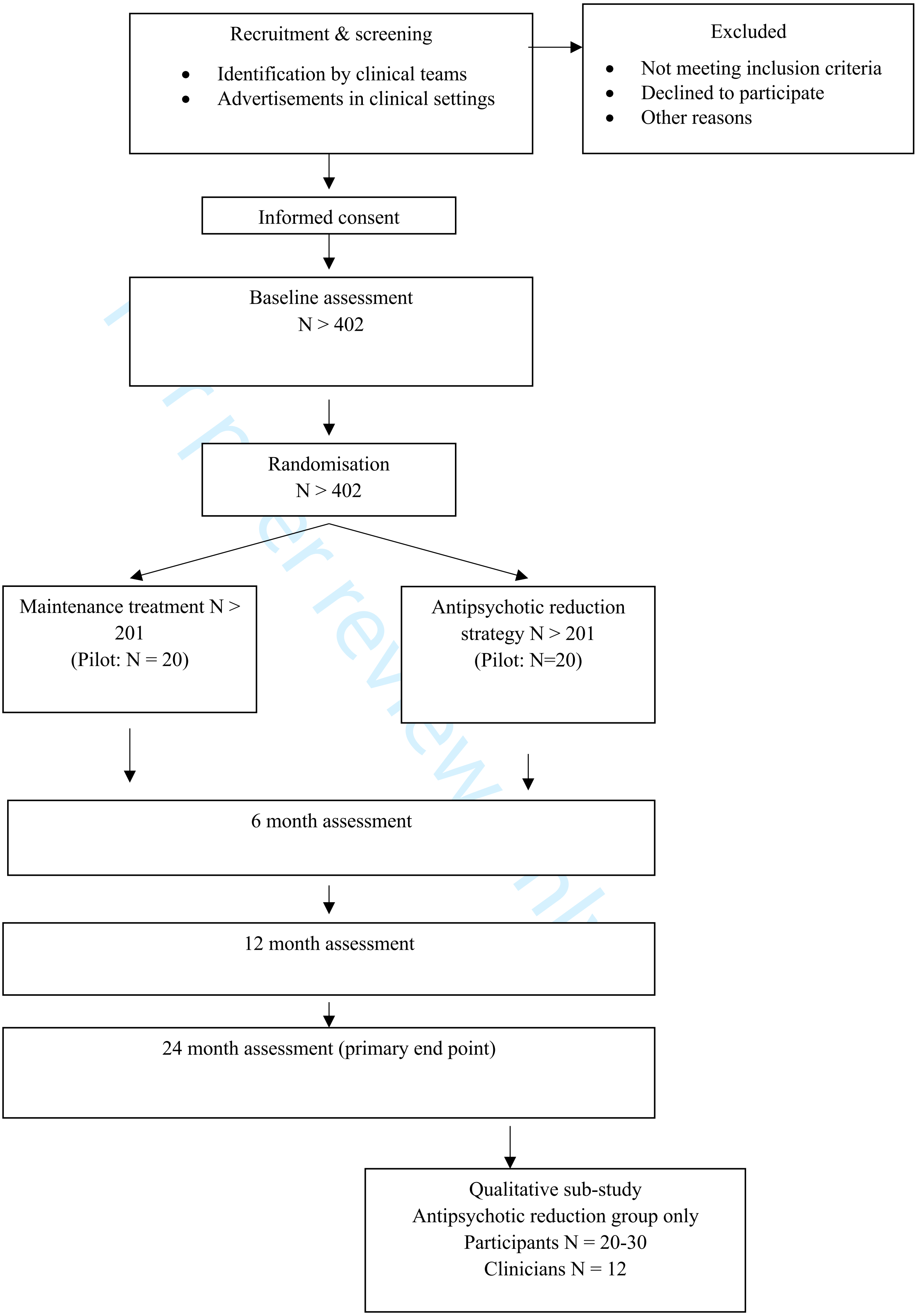

BMJ Open

A randomised controlled trial of gradual antipsychotic reduction and discontinuation in people with schizophrenia and related disorders: The RADAR trial (Research into Antipsychotic Discontinuation and Reduction): Protocol paper

Journal:	*BMJ Open*
Manuscript ID	bmjopen-2019-030912.R1
Article Type:	Protocol
Date Submitted by the Author:	04-Jul-2019

Complete List of Authors:	Moncrieff, Joanna; University College London and North East London mental health trust, Mental Health Sciences<br>Lewis, Glyn; UCL Psychiatric Epidemiology, Mental Health Sciences Unit<br>Freemantle, Nick; University College London, Comprehensive Clinical Trials Unit<br>Johnson, Sonia; UCL, Division of Psychiatry<br>Barnes, Thomas; Imperial College London, Centre for Mental Health<br>Morant, Nicola; University College London,<br>Pinfold, Vanessa; McPin Foundation,<br>Hunter, Rachael; University College London, Research Dept of Primary Care and Population Health<br>Kent, Lyn<br>Smith, Ruth<br>Darton, Katherine<br>Horne, Robert; University College London, School of Pharmacy<br>Crellin, Nadia; UCL, Division of Psychiatry<br>Cooper, Ruth; Queen Mary University of London, Unit for Social and Community Psychiatry<br>Priebe, Stefan; Barts and the London School of Medicine and Dentistry, University of London, Unit of Social and Community Psychiatry

**Primary Subject Heading**:	Mental health
Secondary Subject Heading:	Health services research
Keywords:	antipsychotics, schizophrenia, psychosis, antipsychotic maintenance, schizophrenia, randomised controlled trial

SCHOLARONE™
Manuscripts

A randomised controlled trial of gradual antipsychotic reduction and discontinuation in people with schizophrenia and related disorders: The RADAR trial (Research into Antipsychotic Discontinuation and Reduction)

Protocol paper

Joanna Moncrieff[1,2], Glyn Lewis[1], Nick Freemantle[1], Sonia Johnson[1], Thomas R. E. Barnes[3], Nicola Morant[1], Vanessa Pinfold[4], Rachael Hunter[5], Lyn Kent[6], Ruth Smith[7], Katherine Darton[8], Robert Horne[9], Nadia Crellin[1,2], Ruth Cooper[10], Stefan Priebe[10].

[1]Division of Psychiatry, University College London, London, UK

[2]Research & Development Department, Goodmayes Hospital, North East London NHS Foundation Trust, Essex, UK

[3]Centre for Psychiatry, Imperial College London, London, UK

[4]McPin Foundation, London, UK

[5]Research Department of Primary Care and Population Health, Royal Free Medical School, London, UK

[6]Brentwood, United Kingdom of Great Britain and Northern Ireland

[7]Brighton, United Kingdom of Great Britain and Northern Ireland

[8]London, United Kingdom of Great Britain and Northern Ireland

[9]School of Pharmacy, University College London, London, UK

[10]Newham Centre for Mental Health, Unit for Social and Community Psychiatry, Queen Mary University of London, London, UK

Correspondence to:

Dr Joanna Moncrieff, Division of Psychiatry, Maple House, London, W1T 7NF j.moncrieff@ucl.ac.uk

For peer review only - http://bmjopen.bmj.com/site/about/guidelines.xhtml

For peer review only

Word count: 3850 (not including references, abstract or acknowledgements, author contributions funding and conflict of interest statement).

For peer review only

For peer review only - http://bmjopen.bmj.com/site/about/guidelines.xhtml

ABSTRACT

Introduction

Antipsychotic medication is effective in reducing acute symptoms of psychosis, but it has a range of potentially serious and debilitating adverse effects and is often disliked by patients. It is therefore essential it is only used when benefits outweigh harms. Although multiple trials conducted with people with schizophrenia indicate an increased risk of relapse in the short-term following abrupt antipsychotic discontinuation, there is little evidence about the long-term outcome of a gradual process of reduction and discontinuation on social functioning, relapse and other outcomes.

Methods and analysis

This is a multi-centre, randomised controlled trial involving people with schizophrenia and related disorders who have had more than one episode. Participants are randomised to have a clinically-supervised, gradual reduction of antipsychotic medication, leading to discontinuation when possible, or to continue with maintenance treatment. Blinded follow-up assessments are conducted at 6, 12 and 24 months and the primary outcome is social functioning, measured by the Social Functioning Scale at 24 months. A minimum of 134 evaluable participants provides 90% power to detect a five-point difference, and 206 to detect a four-point difference. Secondary outcomes include severe relapse (admission to hospital) and the study is also intended to detect a minimum 10% difference in severe relapse, which requires 402 participants, assuming a 15% loss to follow-up. Other secondary outcomes include all relapses, as identified by an independent and blinded endpoint committee, symptoms measured by the Positive and Negative Syndrome Scale (PANSS), quality of life, adverse effects, self-rated recovery and neuropsychological measures. Enrolment started in 2016. The trial is scheduled to finish in June 2022.

Ethics and dissemination

Ethical approval was initially obtained on 27/10/2016 (United Kingdom Research Ethics Committee reference 16/LO/1507). Results will be published in peer–reviewed journals and disseminated to the public.

Trial registration ISRCTN 90298520. EudraCT: 2016-000709-36. Pre-results.

For peer review only - http://bmjopen.bmj.com/site/about/guidelines.xhtml

Strengths and limitations

- The trial will be one of the first to provide data on the outcome of a gradual process of antipsychotic reduction and discontinuation in people with schizophrenia and related disorders
- The trial will provide relatively long-term outcome data on social functioning as well as relapse and other outcomes
- There are likely to be some deviations from the planned treatment strategies
- Longer-term follow-up would be desirable and will be initiated in the future

For peer review only

For peer review only - http://bmjopen.bmj.com/site/about/guidelines.xhtml

A randomised controlled trial of gradual antipsychotic reduction and discontinuation in people with schizophrenia and related disorders: The RADAR trial (Research into Antipsychotic Discontinuation and Reduction)

Protocol paper

INTRODUCTION

Schizophrenia and related conditions affect up to 1% of the population (1), and are associated with long-term suffering and disability, premature death, physical illness and high costs to individuals and society (2). Recommended treatment for people with recurrent episodes consists of continuing antipsychotic medication,(3,4). Current guidelines do not recommend attempts at reduction or discontinuation of antipsychotics after the first episode, and in practice, antipsychotic treatment is often 'for life'. In spite of ongoing treatment, many people remain functionally impaired. In one study 25% of people with schizophrenia had severe social disabilities after 15 years, and only 14% had none (5).

The evidence base for long-term antipsychotic treatment consists of studies showing lower relapse rates with maintenance treatment compared with discontinuation (6). However, there are acknowledged methodological problems with these studies (7). In addition, several long-term non-randomised cohorts studies find worse outcomes in people who take continuous treatment compared to those who do not, although confounding by indication is likely to be relevant (8,9). Criticism of randomised controlled trials includes the fact that most focus on relapse and neglect other outcomes. Follow-up is generally short; only six of 65 studies included in a recent meta-analysis by Leucht et al (6) had followed the participants up for more than a year, and longer duration of follow-up was found to be associated with less difference between maintenance and discontinuation (6,10). Moreover, relapse rates may have been inflated by abrupt discontinuation and misidentification of withdrawal-related adverse effects (11,12). Some evidence suggests gradual discontinuation may reduce risk of relapse compared with abrupt discontinuation (13), although this difference was not confirmed in the analysis by Leucht et al (6). However, the average taper of 28 days for gradual medication withdrawal may not have prevented discontinuation-related effects in those people who had been taking antipsychotics for many years.

Long-term antipsychotic medication is associated with potentially serious physical complications, including diabetes, tardive dyskinesia, and cardiovascular disease (14–17).

For peer review only - http://bmjopen.bmj.com/site/about/guidelines.xhtml

Other adverse effects, such as sexual dysfunction, sedation, emotional blunting and akathisia may be debilitating and unpleasant (18–20). Previous research has found that many patients find the adverse effects of antipsychotics burdensome, and would like to try and discontinue the treatment at some stage(21–23).

There are few long-term follow-ups of people from randomised studies of antipsychotic discontinuation. A 7-year follow-up of an 18 month open trial conducted with people with first episode psychosis found that assignment to a gradual antipsychotic reduction programme was associated with better rates of social recovery and equal rates of relapse compared to maintenance treatment (24). Improved levels of neurocognitive performance exhibited by those randomised to antipsychotic reduction at 18 months suggest a possible mechanism for the long-term differences in functional outcome (25). In contrast, recent data from a 10-year follow-up of a one-year, placebo-controlled trial of quetiapine in people with first-episode psychosis reported higher rates of a composite 'poor outcome' in people originally randomised to placebo. There were no differences, however, in overall symptom measures, social functioning or quality of life (26).

Therefore, although continuing antipsychotic treatment has become the norm, it remains unclear whether it has an optimal risk-benefit balance for all people with psychosis or schizophrenia. In particular, more evidence on the effects of gradual reduction of antipsychotics on short and longer term outcomes could inform practice in this area. Effects on social functioning are particularly important to study because of evidence that long-term antipsychotic treatment may impair social functioning, despite improving symptoms or reducing relapse in the short-term (24; 27).

Further studies are currently being conducted in the first-episode psychosis population (28). To date, however, there is no study using gradual and flexible reduction with long-term follow-up in people with more than one episode.

Aims of trial

The current trial was designed to compare the benefits and harms of a gradual programme of dose reduction and discontinuation of antipsychotic treatment, under clinician guidance, with maintenance treatment in people with a diagnosis of schizophrenia or a related disorder. In particular, the study was set up to test whether such a strategy can improve functional outcomes

For peer review only - http://bmjopen.bmj.com/site/about/guidelines.xhtml

in people with recurrent or chronic psychosis while minimising the risk of worsening symptoms or relapse.

METHODS AND ANALYSIS

Study design

The trial consists of an open, parallel group randomised trial with concealed, individual randomisation. Randomisation is conducted through an independent, internet-based system linked with the database (https://www.sealedenvelope.com/randomisation/internet/) with 1:1 allocation. There are no replacements for participants who drop out or otherwise cannot comply with study procedures.

Participants and clinicians are aware of allocation, but outcome assessors are maintained blind to intervention arm as far as possible. This is achieved by nominating separate blinded and unblinded researchers at each site, and ensuring that blinded researchers are not exposed to discussions or written information that would reveal allocation. Following each assessment point blinded research staff record if they suspect they have guessed arm allocation.

The trial lasts for two years, with follow-up assessments conducted at 6, 12 and 24 months post randomisation. The trial design is presented in Figure 1. Ethical approval includes the possibility of conducting a longer-term follow-up, and resources will be sought to support this at a later date.

INSERT FIGURE 1 HERE

Interventions

The antipsychotic reduction protocol was developed after consultation with professionals, academics and the trial's RADAR Lived Experience Advisory Panel (LEAP). Both the antipsychotic reduction and maintenance protocols are administered by treating psychiatrists. For those participants randomised to the antipsychotic medication reduction arm, an individualised reduction schedule is devised by the research team, based on the participant's initial antipsychotic regimen. The dose is reduced incrementally every one or two months, focusing on one drug at a time where participants are taking more than one antipsychotic. The

For peer review only - http://bmjopen.bmj.com/site/about/guidelines.xhtml

rate of reduction varies according to baseline dose, with most schedules aiming for discontinuation within 12 months, but some lasting longer where baseline doses are high. Treating psychiatrists are asked to see the participants who have been randomised to antipsychotic reduction approximately every two months for the duration of the reduction, to adjust the medication regimen and monitor mental state. The participants are offered the option to discontinue antipsychotic medication completely if the reduction progresses well, or to reduce to a very low dose, defined as the equivalent of 2mg of haloperidol a day or less, which is lower than the minimum recommended therapeutic dose for most antipsychotics. This is twice the dose that was defined as a 'low dose' in the Dutch first episode study (24), since some research suggests that doses of antipsychotic medication in people with first episodes should be around half those recommended for people with more established disorders (29,30). Guidance on the antipsychotic reduction strategy stresses the need for flexibility, and includes a suggested protocol for the treatment of adverse reactions to withdrawal or symptom exacerbation. Participants randomised to maintenance treatment are requested not to make major reductions in their dose of antipsychotic medication during the trial period. Increases in dose are permitted within the protocol, as are changes to a different antipsychotic agent at the same equivalent dose and minor dose reductions to address side effects.

Participants are monitored by their care team according to usual practice. Patient records are scrutinised by members of the research team every two months to monitor the progress of the antipsychotic reduction and adherence to the maintenance protocol. Any deviations, such as participants allocated to reduction not starting or halting their reduction, are discussed with the treating clinician. All changes in antipsychotic doses and use of other medications is recorded throughout the study.

Other drug treatment and interventions such as psychological therapies may be used as indicated throughout the trial.

Inclusion and exclusion criteria

The eligibility criteria are detailed in Table 1. They are designed to make the trial as generalizable as possible to routine clinical practice, whilst minimising the risks of antipsychotic reduction in those with a history of posing a serious risk to self or others.

INSERT TABLE 1 HERE

For peer review only - http://bmjopen.bmj.com/site/about/guidelines.xhtml

Outcome measures

The primary outcome is social functioning at two-year follow-up. This will be measured using the Social Functioning Scale (SFS). The scale was developed in 1990, and has good reliability (31,32). It can demonstrate change over time (33–35) and distinguishes different types of antipsychotic treatment (33–36). It also distinguishes between people in remission and those with an ongoing episode (37), people with long versus short DUP (38), people who convert to psychosis versus those who do not in an 'ultra-high risk' cohort (34), and between people who are employed versus unemployed (31). This scale was preferred to the Groningen Social Disability Scale (GSDS) (39), which was used in the Dutch first-episode study, because of the GSDS is long, requires lengthy training and has a strong focus on relationships rather than functioning.

The main measure of the safety of the trial is severe relapse which is defined as admission to acute psychiatric in-patient treatment. This was selected because it is an objective criterion, can be precisely dated and can be obtained through patient records. Previous antipsychotic withdrawal studies have defined relapse in numerous different ways, with no consensus on defining criteria. Therefore, the current trial will also use an expert endpoint committee to assess the presence or absence of relapse based on blinded information from clinical case-notes, based on pre-defined criteria and guidance.

Other outcome measures include: symptoms as measured by the Positive and Negative Syndrome Scale (PANSS) (40), subjective quality of life as measured by the Manchester Short Assessment of quality of life (MANSA) (41), adverse effects of antipsychotics measured by a modified version of the Glasgow Antipsychotic Side-effect Scale (GASS) (42), body weight, sexual dysfunction as measured by the Arizona Sexual Experiences Scale (ASEX) (43), Work Productivity and Activity Impairment- General Health (WPAI) questionnaire (44), neuropsychological function (measured by a brief battery of tests designed for this trial, recovery as measured by the Questionnaire about the Process of Recovery (QPR) (45), ICECAP-A (quality of life), and EQ-5D-5L (quality of life).

In addition, process data will be collected, including dose of antipsychotic medication (in haloperidol equivalents), patient satisfaction as measured by the Client Satisfaction

For peer review only - http://bmjopen.bmj.com/site/about/guidelines.xhtml

Questionnaire CSQ (46) and antipsychotic medication adherence as measured by Medication Adherence Rating Scale MARS-5 (47).

Table 2 shows the time points at which each measure will be completed. All outcome measures are administered by research staff who have received standardised training. Data collected during assessments is stored in a secure online data management system provided by the Sponsor. Participant retention is promoted by sending correspondence and study newsletters to participants facilitate engagement throughout the trial duration.

INSERT TABLE 2 HERE

For peer review only

Recruitment

Participants are recruited from a variety of clinical teams within mental health services across the United Kingdom. Potential participants are identified initially by clinical staff or recruited by advertisements placed in clinical settings. Those who agree are sent further information about the study and then a baseline assessment is arranged after further discussion. Assessments are conducted at the patient's home or in clinical premises according to patient preference.

Risk reduction

The eligibility criteria are designed to exclude people with known high risks of causing harm to themselves or other people. In addition, the gradual nature of the antipsychotic reduction will enable detection and treatment of early signs of relapse. All participants will receive usual care and monitoring of their mental state and behaviour by their clinical team. Those randomised to antipsychotic reduction will have increased contact with a psychiatrist for the duration of the reduction, mirroring usual clinical practice with someone undergoing a significant reduction of medication.

Qualitative assessment

For peer review only - http://bmjopen.bmj.com/site/about/guidelines.xhtml

There will be a qualitative sub-study involving around 20-30 participants from the antipsychotic reduction group and around 12 clinicians with experience of delivering the antipsychotic reduction programme. This study has two aims: to collect data on participant and clinician experience of trial processes, and to explore in detail experiences of antipsychotic reduction and discontinuation from the patient perspective. Participants will be identified towards the end of follow up, using purposive sampling to obtain variation in clinical profile, experience of reduction, completion of the antipsychotic reduction protocol and experience of relapse.

Participants will be interviewed using a semi-structured interview schedule developed in collaboration with the RADAR Lived Experience Advisory Panel (LEAP). This will explore patients' experiences of the intervention and its impact on their mental health and wider lives, the acceptability of antipsychotic reduction, satisfaction with available support, and responses to adverse events. Interviews will be conducted with early participants following the 24-month follow-up interviews so as not to confound the main trial results. Interviews with clinicians will explore their experiences of implementing different aspects of the antipsychotic reduction strategy, relations with the research team, acceptability of medication monitoring procedures, and responses to adverse events. The data will be analysed using thematic analysis.

Statistical analysis

The primary outcome (Social Functioning Scale, SFS) will be analysed using generalised mixed models, accounting for baseline and treatment periods. The principal analysis will be undertaken using the intention to treat population, including all available data, without imputation. Supportive analyses will include analysis of different time periods, analysis using repeated measurements (where all subjects with data at one or more post intervention assessment will be included), exploring potential centre effects and making pessimistic assumptions by group on the patient outcome (to assess the thresholds to the impact of 'missingness') because of the likely pattern of data missing not at random.

The proportions of patients who experience a 'severe relapse' (psychiatric hospital admission) will be compared between the randomised groups at two year follow-up, and survival analysis using Cox constant proportional hazards models including centres as marginal frailty terms

For peer review only - http://bmjopen.bmj.com/site/about/guidelines.xhtml

will also be performed. We will undertake supportive threshold analyses where any loss to follow up will be considered according to extreme data patterns – e.g. assuming all those who were lost in the antipsychotic withdrawal group had a relapse and all those in the maintenance group did not, and thus quantifying the extent of possible effects of missingness and thus the robustness of the main result.

A full statistical analysis plan will be completed and agreed prior to any analysis or unblinding of the data, including planned subgroup analyses and with detailed description of the statistical processes to be used.

Economic analysis

The economic evaluation will evaluate the cost-effectiveness of the antipsychotic reduction strategy compared to maintenance treatment over 24 months. The principal analysis will be the incremental cost per quality adjusted life year gained from the health and social services cost perspective using the EQ-5D-5L to calculate quality adjusted life years (QALYs) as recommended by NICE (48). A secondary analysis will include the cost per capability adjusted life year (CALY) gained of the antipsychotic reduction strategy compared to maintenance treatment over 24 months using the ICECAP-A, argued to have better face validity (49). Additional sensitivity analyses will explore the cost-effectiveness of antipsychotic reduction compared to maintenance capturing the QoL loss associated with antipsychotics side effects using specific disutility values for antipsychotic side effects from the published literature (50).

The Work Productivity and Activity Impairment- General Health (WPAI) questionnaire and associated formula (44) will be used to give a monetary value to impact on employment. Health and social care data costs using published sources will be used to calculate costs for the primary health and social care analysis. A secondary analysis will be conducted from a societal perspective to capture the impact on employment, criminal justice, benefits, family and close others

All costs and outcomes will be discounted in line with NICE guidance at 3.5% per year. Bootstrapping will be used to construct confidence intervals for total mean costs, QALYs and CALYs and to construct cost effectiveness acceptability curves and cost-effectiveness planes. Missing data will be handled in the same way as stated by the statistical analysis plan.

For peer review only - http://bmjopen.bmj.com/site/about/guidelines.xhtml

Sample size calculation

The primary outcome is social functioning, which will be measured by the Social Functioning Scale (31). The literature suggests a minimum difference of between four and five points would be clinically significant, since a difference of four to five points appears to differentiate between patients with good and poor outcomes (37,51,52).

Using a conventional α of 5% (two sided) and taking a SD of 8.8 derived from the literature,(53), a sample size of 134 evaluable participants is required to provide 90% power to identify a five point difference, and a sample of 206 is required to identify a four point difference.

The trial also aimed to be powered to establish the safety of the antipsychotic reduction strategy by powering it to detect whether there is a difference in rates of 'severe' relapse (psychiatric hospitalisation), the principal safety measure, between the intervention arms. We believe that an increased risk of up to 10% would be acceptable to many clinicians and patients if it is balanced by the important outcomes of an improvement in social functioning and reduced side effects. We derived event rates for hospitalisation from those reported in the Leucht et al (6) meta-analysis of antipsychotic discontinuation studies.

We conducted a non-inferiority calculation using a 10% margin of difference. Using a non-inferiority boundary of 10% event rates (severe relapse), with an alpha of 0.05, requires a sample size of 372 for 90% power to exclude a difference of 10% between groups. Adding 15% for attrition brings this up to 402. The lower confidence interval on the absolute scale would exclude a difference of 10% in the situation where non inferiority was achieved. 402 participants provides 90% power to detect a difference of 3.1 points on the primary efficacy outcome (the SFS), including an allowance of 15% for attrition or unexpected challenges to the assumptions.

It is acknowledged that the non-inferiority approach may be unhelpful in these circumstances, as different people may have substantially different views on what is a reasonable non-inferiority boundary. Further, the assumptions required on issues such as the event rate for the non-inferiority assumption are strong, and modest changes in rates can have a substantial impact on the statistical power of the study making the proposed non inferiority boundary

For peer review only - http://bmjopen.bmj.com/site/about/guidelines.xhtml

unhelpful in certain circumstances. Thus the principal aim of the study with regards to severe relapse is to provide a robust estimate of the event rates in each randomised condition, and the difference between them, describing measurement error with appropriate confidence intervals in order to allow individual patients and their clinicians to make an informed decision on treatment options.

PATIENT AND PUBLIC INVOLVEMENT

Testing this research question is a response to a long-standing demand from the service user and carer community for an alternative to long-term antipsychotic medication. Many service users and carers are extremely concerned about the possible long-term side effects of antipsychotic medication. The study has four co-applicants with a patient or public perspective who attend project management meetings; a carer, a service user, an experienced coordinator and researcher, and a staff member of a relevant charity. There is also a LEAP (Lived Experience Advisory Panel), made up of people with expertise in antipsychotic medication through personal use or as a carer for someone with psychosis. It meets regularly to discuss the progress of the study and to contribute to its development, for example, advising on the antipsychotic reduction strategy. The LEAP will be particularly involved in the qualitative study as part of the process evaluation. They will also be involved in providing feedback on study outputs including a regular newsletter and disseminating research results. Results of the study will be disseminated to participants at their request.

MONITORING

The trial is monitored by a representative of the Sponsor according to the agreed trial-specific monitoring plan. A Data Safety and Monitoring Board (DSMB) and Programme Steering Committee provide independent oversight of the trial. The DSMB safeguards the interests of trial participants by assessing the safety and efficacy of the interventions during the trial, and monitoring its conduct and it makes recommendations to the steering committee. There will be no formal interim analysis, but the DSMB will continually review all adverse events data. The trial will be stopped if it is judged that there is a substantial increase in serious adverse events that are likely to be related to the intervention.

SAFETY & ADVERSE EVENTS

For peer review only - http://bmjopen.bmj.com/site/about/guidelines.xhtml

All adverse events are recorded with a description of the event, and are assessed for severity, causality, expectedness and seriousness.

ETHICS AND DISSEMINATION

The trial received approval from the Brent Ethics committee on 27/10/2016. The Research Ethics Committee (REC) reference is 16/LO/1507. The trial was registered with the International Standard Randomised Controlled Trials register on 7/2/2017, registration number: ISRCTN90298520 and with ClinicalTrials.gov on 15/6/2018. All trial data is handled according to the UK Data Protection Act 1998. Substantial protocol amendments are documented and submitted for ethical and regulatory approval prior to implementation.

Written informed consent is obtained prior to participation in the trial, following a full explanation of the aims, methods, anticipated benefits and hazards of the trial. A formal assessment of each participant's capacity to provide consent is completed first. Consent is an ongoing process and researchers ask participants for verbal consent at each follow-up time-point.

Results will be published in peer-reviewed journals and disseminated to the public and the media. All contributors must meet the 'Authorship Criteria', as recommended by The International Committee of Medical Journal Editors.

Current trial status

Recruitment started in March 2016 and will continue until 2020.

DISCUSSION

Better evidence is required about the balance of risks and benefits of a supported programme of antipsychotic reduction and discontinuation under the guidance of a clinician for the many people who have a diagnosis of schizophrenia or recurrent psychoses. The current study will be the first study conducted with people with more than one psychotic episode to employ a gradual and supported method of antipsychotic reduction, to look at a wide range of outcomes

For peer review only - http://bmjopen.bmj.com/site/about/guidelines.xhtml

giving priority to social functioning, and to follow people up for a reasonable duration. Further follow-ups are envisaged after the official end of the trial in order to provide data on longer-term outcomes.

The Dutch first-episode study suggested that long-term outcomes after a period of antipsychotic dose reduction differ from short-term outcomes (24,54). Although relapse rates are increased initially compared with maintenance treatment, over time these equalise. Social functioning, which was not affected in the short-term, was considerably improved by the time of the seven-year follow-up assessment. Social functioning is a measure of independence and personal efficacy. If the current trial results in improvements in social functioning this could reduce an individual's reliance on services and produce significant economic benefits. From the individual's point of view, better social functioning reflects a more fulfilling and socially integrated life. A strategy that can successfully reduce the use of antipsychotic medication is also likely to be associated with health benefits and improvements in quality of life secondary to the reduction of adverse effects.

The outcomes of the current trial will provide good evidence to inform patients and clinicians about the likely consequences of reducing and discontinuing long-term antipsychotic treatment in a gradual manner in a clinical setting. Many patients currently want to consider this option, but existing data on the range of relevant outcomes is limited. Providing further information will facilitate a more collaborative approach to long-term antipsychotic treatment.

For peer review only

For peer review only - http://bmjopen.bmj.com/site/about/guidelines.xhtml

PATIENT CONSENT FOR PUBLICATION: Not required

ACKNOWLEDGEMENTS

This research is sponsored by Priment Clinical Trials Unit, University College London (Sponsor's reference number: 15/0947) and the authors would like to thank members of Priment for their support for the trial. The RADAR research team also acknowledges the support of the National Institute for Health Research Clinical Research Network and North East London NHS Foundation Trust. The research team thank the RADAR Lived Experience Advisory Panel (LEAP) for their expertise, guidance and support. With thanks also to the independent members of the Programme Steering Committee and the Data and Safety Monitoring Board.

AUTHORS' CONTRIBUTIONS

JM developed the original concept for the trial and drafted the protocol. All authors contributed to the trial design and methodology. JM, GL, NF, SJ, TB, NM, VP, RH, LK, RCS, KD, RH & SP are grantholders. NF provided statistical expertise. NC and RC provided trial management and updated the protocol. JM drafted the manuscript, and JM and NC edited subsequent drafts. All authors reviewed and commented on drafts of the paper and approved the final manuscript version. The study Sponsor has contributed to the study design, data collection, management, analysis and interpretation of data.

FUNDING

This report is independent research funded by the National Institute for Health Research (Programme Grants for Applied Research, Research into Antipsychotic Discontinuation And Reduction (RADAR), RP-PG-0514-20004). The views expressed in this publication are those of the author(s) and not necessarily those of the NHS, the National Institute for Health Research or the Department of Health. The NIHR will not have any role during the study execution, analyses, or interpretation of data.

For peer review only - http://bmjopen.bmj.com/site/about/guidelines.xhtml

COMPETING INTERESTS

TREB: In the last three years, TREB has been a member of scientific advisory boards for Otsuka/Lundbeck, Newron Pharmaceuticals and Gedeon Richter and received speaker fees from Janssen.

GL has acted as an expert witness in cases concerning litigation about antidepressants.

No other authors have competing interests to declare.

For peer review only

For peer review only - http://bmjopen.bmj.com/site/about/guidelines.xhtml

REFERENCES

1. McGrath J, Saha S, Chant D, Welham J. Schizophrenia: A Concise Overview of Incidence, Prevalence, and Mortality. *Epidemiol Rev* 2008;14:30(1):67–76. https://doi.org/10.1093/epirev/mxn001

2. McCrone P, Dhanasiri S, Patel A, Knapp.M., Lawton S. Paying the price. the cost of mental health care in England to 2026. London: Kings Fund; 2008.

3. Hasan A, Falkai P, Wobrock T, Lieberman J, Glenthoj B, Gattaz WF, et al. World Federation of Societies of Biological Psychiatry (WFSBP) Guidelines for Biological Treatment of Schizophrenia, Part 2: Update 2012 on the long-term treatment of schizophrenia and management of antipsychotic-induced side effects. *World J Biol Psychiatry* 2013;14(1):2–44. https://doi.org/10.3109/15622975.2012.739708

4. National Institute for Health and Care Excellence. Psychosis and schizophrenia in adults: treatment and management. London: NICE; 2014.

5. Wiersma D, Wanderling J, Dragomirecka E, Ganev K, Harrison G, an der HW, et al. Social disability in schizophrenia: its development and prediction over 15 years in incidence cohorts in six European centres. *Psychol Med* 2000;30(5):1155-67. https://doi.org/10.1017/S0033291799002627

6. Leucht S, Tardy M, Komossa K, Heres S, Kissling W, Davis JM. Maintenance treatment with antipsychotic drugs for schizophrenia. *Cochrane Database Syst Rev* 2012;5:CD008016. https://doi.org/10.1002/14651858.CD008016.pub2

7. Leucht S, Heres S, Hamann J, Kane JM. Methodological Issues in Current Antipsychotic Drug Trials. *Schizophr Bull* 2008;9;34(2):275–85. https://doi.org/10.1093/schbul/sbm159

8. Harrow M, Jobe TH, Faull RN. Do all schizophrenia patients need antipsychotic treatment continuously throughout their lifetime? A 20-year longitudinal study. *Psychol Med* 2012;42(10):2145–55. https://doi.org/10.1017/S0033291712000220

9. Moilanen JM, Haapea M, Jääskeläinen E, Veijola JM, Isohanni MK, Koponen HJ, et al. Long-term antipsychotic use and its association with outcomes in schizophrenia – the Northern Finland Birth Cohort 1966. *Eur Psychiatry* 2016;36:7–14. https://doi.org/10.1016/j.eurpsy.2016.03.002

10. Gilbert PL, Harris MJ, McAdams LA, Jeste DV. Neuroleptic withdrawal in schizophrenic patients. A review of the literature. *Arch Gen Psychiatry* 1995;52(3):173-88. https://doi.org/10.1001/archpsyc.1995.03950150005001

11. Baldessarini RJ, Viguera AC. Neuroleptic withdrawal in schizophrenic patients. *Arch Gen Psychiatry* 1995;52(3):189-92. https://doi.org/10.1001/archpsyc.1995.03950150021002

12. Whitaker R. Anatomy of an Epidemic. New York: Crown Publishers; 2010.

13. Viguera AC, Baldessarini RJ, Hegarty JD, van Kammen DP, Tohen M. Clinical risk following abrupt and gradual withdrawal of maintenance neuroleptic treatment. *Arch Gen Psychiatry* 1997;54(1):49–55. https://doi.org/10.1001/archpsyc.1997.01830130055011

For peer review only - http://bmjopen.bmj.com/site/about/guidelines.xhtml

14. Osborn DPJ, Levy G, Nazareth I, Petersen I, Islam A, King MB. Relative Risk of Cardiovascular and Cancer Mortality in People With Severe Mental Illness From the United Kingdom's General Practice Research Database. *Arch Gen Psychiatry* 2007;64(2):242-9. https://doi.org/10.1001/archpsyc.64.2.242

15. Ray WA, Chung CP, Murray KT, Hall K, Stein CM. Atypical antipsychotic drugs and the risk of sudden cardiac death. *N Engl J Med* 2009;15;360(3):225-35. https://doi.org/10.1056/NEJMoa0806994

16. Yood MU, deLorenze G, Quesenberry CP, Oliveria SA, Tsai A-L, Willey VJ, et al. The incidence of diabetes in atypical antipsychotic users differs according to agent-results from a multisite epidemiologic study. *Pharmacoepidemiol Drug Saf* 2009;18(9):791–9. https://doi.org/10.1002/pds.1781

17. Correll CU, Schenk EM. Tardive dyskinesia and new antipsychotics. *Curr Opin Psychiatry* 2008;21(2):151-6. https://doi.org/10.1097/YCO.0b013e3282f53132

18. Moncrieff J, Cohen D, Mason JP. The subjective experience of taking antipsychotic medication: a content analysis of Internet data. *Acta Psychiatr Scand* 2009;120(2):102–11. https://doi.org/10.1111/j.1600-0447.2009.01356.x

19. Barbui C, Nose M, Bindman J, Schene A, Becker T, Mazzi MA, et al. Sex Differences in the Subjective Tolerability of Antipsychotic Drugs. *J Clin Psychopharmacol* 2005;25(6):521–6. https://doi.org/10.1097/01.jcp.0000185423.15891.02

20. Hatano M, Kamei H, Kato A, Takeuchi I, Hanya M, Uno J, et al. Assessment of the Latent Adverse Events of Antipsychotic Treatment Using a Subjective Questionnaire in Japanese Patients with Schizophrenia. *Clin Psychopharmacol Neurosci* 2017;31;15(2):132–7. https://doi.org/10.9758/cpn.2017.15.2.132

21. Williams K, Pinfold V. Side Effects: Mental health service users experiences of the side effects of antipsychotic medication. London: Rethink; 2006.

22. Read J. Psychiatric Drugs: Key issues and service user perspectives. Basingstoke, Hants: Palgrave Macmillan; 2009.

23. Morant N, Azam K, Johnson S, Moncrieff J. The least worst option: user experiences of antipsychotic medication and lack of involvement in medication decisions in a UK community sample. *J Ment Health* 2018;4;27(4):322–8. https://doi.org/10.1080/09638237.2017.1370637

24. Wunderink L, Nieboer RM, Wiersma D, Sytema S, Nienhuis FJ. Recovery in Remitted First-Episode Psychosis at 7 Years of Follow-up of an Early Dose Reduction/Discontinuation or Maintenance Treatment Strategy: Long-term Follow-up of a 2-Year Randomized Clinical Trial. *JAMA Psychiatry* 2013;1;70(9):913. https://doi.org/10.1001/jamapsychiatry.2013.19

25. Faber G, Smid HGOM, Van Gool AR, Wunderink L, Wiersma D, van den Bosch RJ. Neurocognition and recovery in first episode psychosis. *Psychiatry Res* 2011;188(1):1–6. https://doi.org/10.1016/j.psychres.2010.11.010

26. Hui CLM, Honer WG, Lee EHM, Chang WC, Chan SKW, Chen ESM, et al. Long-term effects of discontinuation from antipsychotic maintenance following first-episode schizophrenia and related disorders: a 10 year follow-up of a randomised, double-blind trial. *Lancet Psychiatry* 2018;5(5):432–42. https://doi.org/10.1016/S2215-0366(18)30090-7

For peer review only - http://bmjopen.bmj.com/site/about/guidelines.xhtml

27. Wils RS, Gotfredsen DR, Hjorthøj C, Austin SF, Albert N, Secher RG, et al. Antipsychotic medication and remission of psychotic symptoms 10 years after a first-episode psychosis. *Schizophr Res* 2017;182:42-48. doi: 10.1016/j.schres.2016.10.030.

28. Stürup AE, Jensen HD, Dolmer S, Birk M, Albert N, Nielsen M, et al. TAILOR – tapered discontinuation versus maintenance therapy of antipsychotic medication in patients with newly diagnosed schizophrenia or persistent delusional disorder in remission of psychotic symptoms: study protocol for a randomized clinical trial. *Trials* [Internet]. 2017 Dec [cited 2019 Feb 18];18(1). Available from: http://trialsjournal.biomedcentral.com/articles/10.1186/s13063-017-2172-4

29. McEvoy JP, Hogarty GE, Steingard S. Optimal dose of neuroleptic in acute schizophrenia. A controlled study of the neuroleptic threshold and higher haloperidol dose. *Arch Gen Psychiatry* 1991;48(8):739–45. https://doi.org/10.1001/archpsyc.1991.01810320063009

30. Oosthuizen P, Emsley RA, Turner J, Keyter N. Determining the optimal dose of haloperidol in first-episode psychosis. *J Psychopharmacol (Oxf)* 2001;15(4):251–5. https://doi.org/10.1177/026988110101500403

31. Birchwood M, Smith J, Cochrane R, Wetton S, Copestake S. The Social Functioning Scale. The Development and Validation of a New Scale of Social Adjustment for use in Family Intervention Programmes with Schizophrenic Patients. *Br J Psychiatry* 1990;157(06):853–9. https://doi.org/10.1192/bjp.157.6.853

32. Vázquez Morejón AJ, Jiménez Ga-Bóveda R. Social Functioning Scale: new contributions concerning its psychometric characteristics in a Spanish adaptation. *Psychiatry Res* 2000;93(3):247–56. https://doi.org/10.1016/S0165-1781(99)00123-7

33. Ciudad A, Olivares JM, Bousoño M, Gómez JC, Álvarez E. Improvement in social functioning in outpatients with schizophrenia with prominent negative symptoms treated with olanzapine or risperidone in a 1 year randomized, open-label trial. *Prog Neuropsychopharmacol Biol Psychiatry* 2006;30(8):1515–22. https://doi.org/10.1016/j.pnpbp.2006.05.010

34. Jang JH, Shin NY, Shim G, Park HY, Kim E, Jang G-E, et al. Longitudinal Patterns of Social Functioning and Conversion to Psychosis in Subjects at Ultra-High Risk. *Aust N Z J Psychiatry* 2011;45(9):763–70. https://doi.org/10.3109/00048674.2011.595684

35. Sheridan AJ, Drennan J, Coughlan B, O'Keeffe D, Frazer K, Kemple M, et al. Improving social functioning and reducing social isolation and loneliness among people with enduring mental illness: Report of a randomised controlled trial of supported socialisation. *Int J Soc Psychiatry* 2015;61(3):241–50. https://doi.org/10.1177/0020764014540150

36. Koshikawa Y, Takekita Y, Kato M, Sakai S, Onohara A, Sunada N, et al. The Comparative Effects of Risperidone Long-Acting Injection and Paliperidone Palmitate on Social Functioning in Schizophrenia: A 6-Month, Open-Label, Randomized Controlled Pilot Trial. *Neuropsychobiology* 2016;73(1):35–42. https://doi.org/10.1159/000442209

37. Jaracz K, Górna K, Kiejda J, Grabowska-Fudala B, Jaracz J, Suwalska A, et al. Psychosocial functioning in relation to symptomatic remission: A longitudinal study of first episode schizophrenia. *Eur Psychiatry* 2015;30(8):907–13. https://doi.org/10.1016/j.eurpsy.2015.08.001

For peer review only - http://bmjopen.bmj.com/site/about/guidelines.xhtml

38. Barnes TRE, Leeson VC, Mutsatsa SH, Watt HC, Hutton SB, Joyce EM. Duration of untreated psychosis and social function: 1-year follow-up study of first-episode schizophrenia. *Br J Psychiatry* 2008;193(03):203–9. https://doi.org/10.1192/bjp.bp.108.049718

39. Wiersma D, DeJong A, Ormel J. The Groningen Social Disabilities Schedule: development, relationship with I.C.I.D.H., and psychometric properties. *Int J Rehabil Res* 1988;11(3):213-24.

40. Kay SR, Fiszbein A, Opler LA. The Positive and Negative Syndrome Scale (PANSS) for Schizophrenia. *Schizophr Bull* 1987;1;13(2):261–76. https://doi.org/10.1093/schbul/13.2.261

41. Priebe S, Huxley P, Knight S, Evans S. Application and Results of the Manchester Short Assessment of Quality of Life (Mansa). *Int J Soc Psychiatry* 1999;45(1):7–12. https://doi.org/10.1177/002076409904500102

42. Waddell L, Taylor M. A new self-rating scale for detecting atypical or second-generation antipsychotic side effects. *J Psychopharmacol (Oxf)* 2008;22(3):238–43. https://doi.org/10.1177/0269881107087976

43. McGahuey CA, Gelenberg AJ, Laukes CA, Moreno FA, Delgado PL, McKnight KM, et al. The Arizona Sexual Experience Scale (ASEX): reliability and validity. *J Sex Marital Ther* 2000 Jan;26(1):25-40. https://doi.org/10.1080/009262300278623

44. Reilly MC, Zbrozek AS, Dukes EM. The validity and reproducibility of a work productivity and activity impairment instrument. *Pharmacoeconomics* 1993;4(5):353–65. https://doi.org/10.2165/00019053-199304050-00006

45. Law H, Neil ST, Dunn G, Morrison AP. Psychometric properties of the Questionnaire about the Process of Recovery (QPR). *Schizophr Res* 2014;156(2–3):184–9. https://doi.org/10.1016/j.schres.2014.04.011

46. Atkisson CC, Zwick R. The Client Satisfaction Questionnaire: Psychometric properties and correlations with service utilisation and psychotherapy outcome. *Evaluation and Program Planning* 1982;5(3):233-7. https://doi.org/10.1016/0149-7189(82)90074-X

47. Mahler C, Hermann K, Horne R, Ludt S, Haefeli WE, Szecsenyi J, et al. Assessing reported adherence to pharmacological treatment recommendations. Translation and evaluation of the Medication Adherence Report Scale (MARS) in Germany. *J Eval Clin Pract* 2010;16(3):574-9. https://doi.org/10.1111/j.1365-2753.2009.01169.x

48. EuroQol Group. EuroQol--a new facility for the measurement of health-related quality of life. *Health Policy* 1990;16(3):199-208. https://doi.org/10.1016/0168-8510(90)90421-9

49. Brazier J, Connell J, Papaioannou D, Mukuria C, Mulhern B, Peasgood T, et al. A systematic review, psychometric analysis and qualitative assessment of generic preference-based measures of health in mental health populations and the estimation of mapping functions from widely used specific measures. *Health Technol Assess* 2014;18(34):vii-xxv,1. https://doi.org/10.3310/hta18340

50. Briggs A, Wild D, Lees M, Reaney M, Dursun S, Parry D, et al. Impact of schizophrenia and schizophrenia treatment-related adverse events on quality of life: direct utility elicitation. *Health Qual Life Outcomes* 2008;6(1):105. https://doi.org/10.1186/1477-7525-6-105

For peer review only - http://bmjopen.bmj.com/site/about/guidelines.xhtml

51. Hellvin T, Sundet K, Vaskinn A, Simonsen C, Ueland T, Andreassen OA, et al. Validation of the Norwegian version of the Social Functioning Scale (SFS) for schizophrenia and bipolar disorder: Validation of the SFS (Norwegian version). *Scand J Psychol* 2010;51(6):525–33. https://doi.org/10.1111/j.1467-9450.2010.00839.x

52. Leeson VC, Harrison I, Ron MA, Barnes TRE, Joyce EM. The Effect of Cannabis Use and Cognitive Reserve on Age at Onset and Psychosis Outcomes in First-Episode Schizophrenia. *Schizophr Bull* 2012;1;38(4):873–80. https://doi.org/10.1093/schbul/sbq153

53. Slupczynska-Kossobudzka E, Boguszewska L. Effects of Community Mobile Team Intervention in the Drewnica Hospital Catchment Area. 1. Patient Outcome. *Int J Soc Psychiatry* 1999;45(3):207–15. https://doi.org/10.1177/002076409904500308

54. Wunderink L, Nienhuis FJ, Sytema S, Slooff CJ, Knegtering R, Wiersma D. Guided discontinuation versus maintenance treatment in remitted first-episode psychosis: relapse rates and functional outcome. *J Clin Psychiatry* 2007;68(5):654-61. https://doi.org/10.4088/JCP.v68n0502

For peer review only - http://bmjopen.bmj.com/site/about/guidelines.xhtml

Table 1. Eligibility criteria

**Inclusion criteria**	
1.	Aged 18 years or older
2.	A clinical and/or ICD10 diagnosis of schizophrenia, schizoaffective disorder, delusional disorder or other non-affective psychosis
3.	More than one previous episode or psychotic exacerbation, or a single episode lasting more than one year
4.	Prescribed continuing antipsychotic medication
**Exclusion criteria**	
1.	Lack of capacity to consent to the trial.
2.	Insufficient command of spoken English to understand trial procedures
3.	Subject to a section of the Mental Health Act (MHA) that includes a requirement to take antipsychotic medication
4.	Clinician considers there will be a serious risk of harm to self or others
5.	Admitted to hospital or treated by a Home Treatment or Crisis Team within the last month
6.	Women who have a confirmed pregnancy
7.	Women who are breast-feeding
8.	Involvement in another Investigational Medicinal Product (IMP) trial

For peer review only

For peer review only - http://bmjopen.bmj.com/site/about/guidelines.xhtml

Table 2. Time points at which different outcomes will be assessed

*A = Demographic information (selected sections including weight and use of illicit drugs and alcohol); B = Diagnosis (established from clinical records); C = Social Functioning Scale (SFS); D = Positive and Negative Syndrome Scale (PANSS); E = Glasgow Antipsychotic Side-effect Scale (GASS); F = Client Satisfaction Questionnaire (CSQ-8); G = Manchester Short Assessment of quality of life (MANSA); H = Neuropsychological function tests; I = Medication Adherence Rating Scale (MARS-5); J = Relapse questionnaire ; K = Serious Adverse Events; L = EQ-5D-5L; M = ICECAP-A; N = Client Service Receipt Inventory ; O = Work Productivity and Activity Questionnaire; P = Schedule for economic data from patient records; Q = Questionnaire about the Process of Recovery (QPR); R = Arizona Sexual Experiences Scale (ASEX).

	**Baseline**	**Follow-up**				
**Visit Number**	**1**	**2 Pilot trial 3-month data collection**	**3 6-month follow-up**	**4 12-month follow-up**	**5 24-month follow-up**	**6 Qualitative evaluation**
**Informed Consent**	X					X
**Eligibility determination**	X					
**Protocol Assessments**	A-I, L-O, Q, R	K,	C-G, I-O, Q, R	C-O,Q, R	C- R	Indicative topic guide with sample of participants and psychiatrists
**Randomisation**	X					
**IMP administration**	X	X	X	X	X	
**Adverse Events review**	X	X	X	X	X	
**Medical notes review for prescribing information and fidelity to intervention protocols**	X	X	X	X	X	
**Concomitant Medication review**	X	X	X	X	X	

For peer review only - http://bmjopen.bmj.com/site/about/guidelines.xhtml

Figure 1. Trial Flow Chart

For peer review only

For peer review only - http://bmjopen.bmj.com/site/about/guidelines.xhtml

Figure 1. Trial Flow Chart

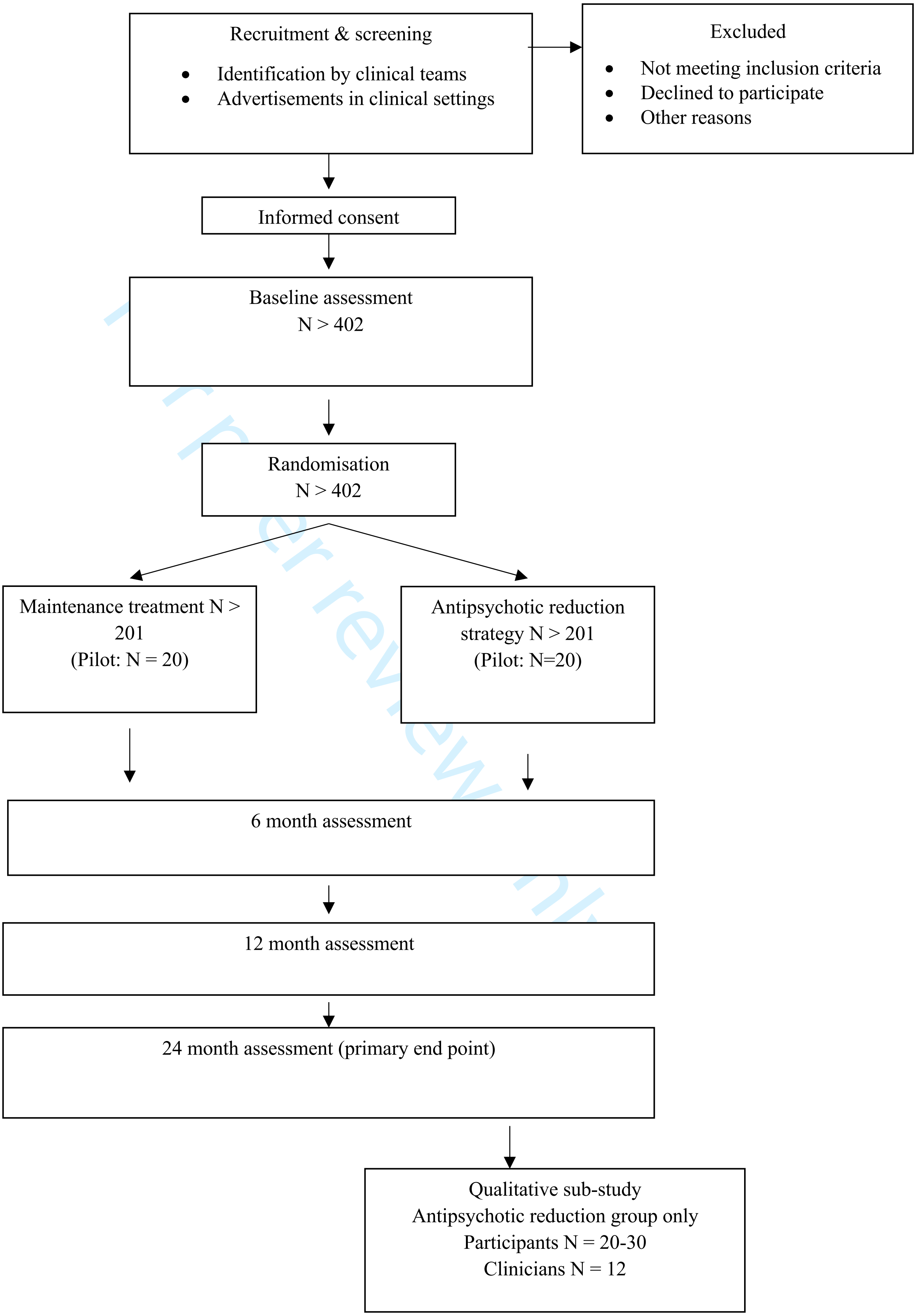

SPIRIT

STANDARD PROTOCOL ITEMS: RECOMMENDATIONS FOR INTERVENTIONAL TRIALS

SPIRIT 2013 Checklist: Recommended items to address in a clinical trial protocol and related documents*

Section/item	Item No	Description	Addressed on page number
**Administrative information**			
Title	1	Descriptive title identifying the study design, population, interventions, and, if applicable, trial acronym	1
Trial registration	2a	Trial identifier and registry name. If not yet registered, name of intended registry	2
	2b	All items from the World Health Organization Trial Registration Data Set	
Protocol version	3	Date and version identifier	
Funding	4	Sources and types of financial, material, and other support	16
Roles and responsibilities	5a	Names, affiliations, and roles of protocol contributors	1/16
	5b	Name and contact information for the trial sponsor	18
	5c	Role of study sponsor and funders, if any, in study design; collection, management, analysis, and interpretation of data; writing of the report; and the decision to submit the report for publication, including whether they will have ultimate authority over any of these activities	17
	5d	Composition, roles, and responsibilities of the coordinating centre, steering committee, endpoint adjudication committee, data management team, and other individuals or groups overseeing the trial, if applicable (see Item 21a for data monitoring committee)	18

For peer review only

Introduction

Background and rationale	6a	Description of research question and justification for undertaking the trial, including summary of relevant studies (published and unpublished) examining benefits and harms for each intervention	4/5
	6b	Explanation for choice of comparators	4/5
Objectives	7	Specific objectives or hypotheses	5
Trial design	8	Description of trial design including type of trial (eg, parallel group, crossover, factorial, single group), allocation ratio, and framework (eg, superiority, equivalence, noninferiority, exploratory)	6/12

Methods: Participants, interventions, and outcomes

Study setting	9	Description of study settings (eg, community clinic, academic hospital) and list of countries where data will be collected. Reference to where list of study sites can be obtained	7
Eligibility criteria	10	Inclusion and exclusion criteria for participants. If applicable, eligibility criteria for study centres and individuals who will perform the interventions (eg, surgeons, psychotherapists)	7/8
Interventions	11a	Interventions for each group with sufficient detail to allow replication, including how and when they will be administered	6/7
	11b	Criteria for discontinuing or modifying allocated interventions for a given trial participant (eg, drug dose change in response to harms, participant request, or improving/worsening disease)	6/7
	11c	Strategies to improve adherence to intervention protocols, and any procedures for monitoring adherence (eg, drug tablet return, laboratory tests)	6/7
	11d	Relevant concomitant care and interventions that are permitted or prohibited during the trial	6/7
Outcomes	12	Primary, secondary, and other outcomes, including the specific measurement variable (eg, systolic blood pressure), analysis metric (eg, change from baseline, final value, time to event), method of aggregation (eg, median, proportion), and time point for each outcome. Explanation of the clinical relevance of chosen efficacy and harm outcomes is strongly recommended	8/9
Participant timeline	13	Time schedule of enrolment, interventions (including any run-ins and washouts), assessments, and visits for participants. A schematic diagram is highly recommended (see Figure)	6

For peer review only - http://bmjopen.bmj.com/site/about/guidelines.xhtml

Sample size	14	Estimated number of participants needed to achieve study objectives and how it was determined, including clinical and statistical assumptions supporting any sample size calculations	12/13
Recruitment	15	Strategies for achieving adequate participant enrolment to reach target sample size	11
**Methods: Assignment of interventions (for controlled trials)**			
Allocation:			
Sequence generation	16a	Method of generating the allocation sequence (eg, computer-generated random numbers), and list of any factors for stratification. To reduce predictability of a random sequence, details of any planned restriction (eg, blocking) should be provided in a separate document that is unavailable to those who enrol participants or assign interventions	6
Allocation concealment mechanism	16b	Mechanism of implementing the allocation sequence (eg, central telephone; sequentially numbered, opaque, sealed envelopes), describing any steps to conceal the sequence until interventions are assigned	6
Implementation	16c	Who will generate the allocation sequence, who will enrol participants, and who will assign participants to interventions	6
Blinding (masking)	17a	Who will be blinded after assignment to interventions (eg, trial participants, care providers, outcome assessors, data analysts), and how	6
	17b	If blinded, circumstances under which unblinding is permissible, and procedure for revealing a participant's allocated intervention during the trial	Na
**Methods: Data collection, management, and analysis**			
Data collection methods	18a	Plans for assessment and collection of outcome, baseline, and other trial data, including any related processes to promote data quality (eg, duplicate measurements, training of assessors) and a description of study instruments (eg, questionnaires, laboratory tests) along with their reliability and validity, if known. Reference to where data collection forms can be found, if not in the protocol	9
	18b	Plans to promote participant retention and complete follow-up, including list of any outcome data to be collected for participants who discontinue or deviate from intervention protocols	9

Data management	19	Plans for data entry, coding, security, and storage, including any related processes to promote data quality (eg, double data entry; range checks for data values). Reference to where details of data management procedures can be found, if not in the protocol	9
Statistical methods	20a	Statistical methods for analysing primary and secondary outcomes. Reference to where other details of the statistical analysis plan can be found, if not in the protocol	12/13/14
	20b	Methods for any additional analyses (eg, subgroup and adjusted analyses)	12/13/14
	20c	Definition of analysis population relating to protocol non-adherence (eg, as randomised analysis), and any statistical methods to handle missing data (eg, multiple imputation)	12/13/14
**Methods: Monitoring**			
Data monitoring	21a	Composition of data monitoring committee (DMC); summary of its role and reporting structure; statement of whether it is independent from the sponsor and competing interests; and reference to where further details about its charter can be found, if not in the protocol. Alternatively, an explanation of why a DMC is not needed	15
	21b	Description of any interim analyses and stopping guidelines, including who will have access to these interim results and make the final decision to terminate the trial	15
Harms	22	Plans for collecting, assessing, reporting, and managing solicited and spontaneously reported adverse events and other unintended effects of trial interventions or trial conduct	15
Auditing	23	Frequency and procedures for auditing trial conduct, if any, and whether the process will be independent from investigators and the sponsor	15
**Ethics and dissemination**			
Research ethics approval	24	Plans for seeking research ethics committee/institutional review board (REC/IRB) approval	15
Protocol amendments	25	Plans for communicating important protocol modifications (eg, changes to eligibility criteria, outcomes, analyses) to relevant parties (eg, investigators, REC/IRBs, trial participants, trial registries, journals, regulators)	15/16

For peer review only - http://bmjopen.bmj.com/site/about/guidelines.xhtml

Consent or assent	26a	Who will obtain informed consent or assent from potential trial participants or authorised surrogates, and how (see Item 32)	16
	26b	Additional consent provisions for collection and use of participant data and biological specimens in ancillary studies, if applicable	Na
Confidentiality	27	How personal information about potential and enrolled participants will be collected, shared, and maintained in order to protect confidentiality before, during, and after the trial	16
Declaration of interests	28	Financial and other competing interests for principal investigators for the overall trial and each study site	18/19
Access to data	29	Statement of who will have access to the final trial dataset, and disclosure of contractual agreements that limit such access for investigators	Na
Ancillary and post-trial care	30	Provisions, if any, for ancillary and post-trial care, and for compensation to those who suffer harm from trial participation	Na
Dissemination policy	31a	Plans for investigators and sponsor to communicate trial results to participants, healthcare professionals, the public, and other relevant groups (eg, via publication, reporting in results databases, or other data sharing arrangements), including any publication restrictions	16
	31b	Authorship eligibility guidelines and any intended use of professional writers	16
	31c	Plans, if any, for granting public access to the full protocol, participant-level dataset, and statistical code	Na
**Appendices**			
Informed consent materials	32	Model consent form and other related documentation given to participants and authorised surrogates	Supplementary
Biological specimens	33	Plans for collection, laboratory evaluation, and storage of biological specimens for genetic or molecular analysis in the current trial and for future use in ancillary studies, if applicable	Na

*It is strongly recommended that this checklist be read in conjunction with the SPIRIT 2013 Explanation & Elaboration for important clarification on the items. Amendments to the protocol should be tracked and dated. The SPIRIT checklist is copyrighted by the SPIRIT Group under the Creative Commons "Attribution-NonCommercial-NoDerivs 3.0 Unported" license.

For peer review only - http://bmjopen.bmj.com/site/about/guidelines.xhtml

BMJ Open

A randomised controlled trial of gradual antipsychotic reduction and discontinuation in people with schizophrenia and related disorders: The RADAR trial (Research into Antipsychotic Discontinuation and Reduction) Protocol paper

Journal:	*BMJ Open*
Manuscript ID	bmjopen-2019-030912.R2
Article Type:	Protocol
Date Submitted by the Author:	20-Aug-2019

Complete List of Authors:	Moncrieff, Joanna; University College London and North East London mental health trust, Mental Health Sciences<br>Lewis, Glyn; UCL Psychiatric Epidemiology, Mental Health Sciences Unit<br>Freemantle, Nick; University College London, Comprehensive Clinical Trials Unit<br>Johnson, Sonia; UCL, Division of Psychiatry<br>Barnes, Thomas; Imperial College London, Centre for Mental Health<br>Morant, Nicola; University College London,<br>Pinfold, Vanessa; McPin Foundation,<br>Hunter, Rachael; University College London, Research Dept of Primary Care and Population Health<br>Kent, Lyn; Independent consultant<br>Smith, Ruth; Independent consultant<br>Darton, Katherine; Independent consultant<br>Horne, Robert; University College London, School of Pharmacy<br>Crellin, Nadia; UCL, Division of Psychiatry<br>Cooper, Ruth; Queen Mary University of London, Unit for Social and Community Psychiatry<br>Priebe, Stefan; Barts and the London School of Medicine and Dentistry, University of London, Unit of Social and Community Psychiatry

**Primary Subject Heading**:	Mental health
Secondary Subject Heading:	Health services research
Keywords:	antipsychotics, schizophrenia, psychosis, antipsychotic maintenance, schizophrenia, randomised controlled trial

SCHOLARONE™
Manuscripts

A randomised controlled trial of gradual antipsychotic reduction and discontinuation in people with schizophrenia and related disorders: The RADAR trial (Research into Antipsychotic Discontinuation and Reduction)

Protocol paper

Joanna Moncrieff[1,2], Glyn Lewis[1], Nick Freemantle[1], Sonia Johnson[1], Thomas R. E. Barnes[3], Nicola Morant[1], Vanessa Pinfold[4], Rachael Hunter[5], Lyn Kent[6], Ruth Smith[7], Katherine Darton[8], Robert Horne[9], Nadia Crellin[1,2], Ruth Cooper[10], Stefan Priebe[10].

[1]Division of Psychiatry, University College London, London, UK

[2]Research & Development Department, Goodmayes Hospital, North East London NHS Foundation Trust, Essex, UK

[3]Centre for Psychiatry, Imperial College London, London, UK

[4]McPin Foundation, London, UK

[5]Research Department of Primary Care and Population Health, Royal Free Medical School, London, UK

[6] Independent consultant, Brentwood, UK

[7] Independent consultant, Brighton, UK

[8] Independent consultant, London, UK

[9]School of Pharmacy, University College London, London, UK

[10]Newham Centre for Mental Health, Unit for Social and Community Psychiatry, Queen Mary University of London, London, UK

Correspondence to:

Dr Joanna Moncrieff, Division of Psychiatry, Maple House, London, W1T 7NF j.moncrieff@ucl.ac.uk

For peer review only - http://bmjopen.bmj.com/site/about/guidelines.xhtml

Word count: 3650 (not including references, abstract or acknowledgements, author contributions funding and conflict of interest statement).

For peer review only

For peer review only - http://bmjopen.bmj.com/site/about/guidelines.xhtml

ABSTRACT

Introduction

Antipsychotic medication is effective in reducing acute symptoms of psychosis, but it has a range of potentially serious and debilitating adverse effects and is often disliked by patients. It is therefore essential it is only used when benefits outweigh harms. Although multiple trials conducted with people with schizophrenia indicate an increased risk of relapse in the short-term following abrupt antipsychotic discontinuation, there is little evidence about the long-term outcome of a gradual process of reduction and discontinuation on social functioning, relapse and other outcomes.

Methods and analysis

This is a multi-centre, randomised controlled trial involving people with schizophrenia and related disorders who have had more than one episode. Participants are randomised to have a clinically-supervised, gradual reduction of antipsychotic medication, leading to discontinuation when possible, or to continue with maintenance treatment. Blinded follow-up assessments are conducted at 6, 12 and 24 months and the primary outcome is social functioning, measured by the Social Functioning Scale at 24 months. A minimum of 134 evaluable participants provides 90% power to detect a five-point difference, and 206 to detect a four-point difference. Secondary outcomes include severe relapse (admission to hospital) and the study is also intended to detect a minimum 10% difference in severe relapse, which requires 402 participants, assuming a 15% loss to follow-up. Other secondary outcomes include all relapses, as identified by an independent and blinded endpoint committee, symptoms measured by the Positive and Negative Syndrome Scale (PANSS), quality of life, adverse effects, self-rated recovery and neuropsychological measures. Enrolment started in 2016. The trial is scheduled to finish in June 2022.

Ethics and dissemination

Ethical approval was initially obtained on 27/10/2016 (United Kingdom Research Ethics Committee reference 16/LO/1507). Results will be published in peer–reviewed journals and disseminated to the public.

Trial registration ISRCTN 90298520. EudraCT: 2016-000709-36. Pre-results.

For peer review only - http://bmjopen.bmj.com/site/about/guidelines.xhtml

Strengths and limitations

- The trial will be one of the first to provide data on the outcome of a gradual process of antipsychotic reduction and discontinuation in people with schizophrenia and related disorders
- The trial will provide relatively long-term outcome data on social functioning as well as relapse and other outcomes
- There are likely to be some deviations from the planned treatment strategies
- Longer-term follow-up would be desirable and will be initiated in the future

For peer review only

For peer review only - http://bmjopen.bmj.com/site/about/guidelines.xhtml

A randomised controlled trial of gradual antipsychotic reduction and discontinuation in people with schizophrenia and related disorders: The RADAR trial (Research into Antipsychotic Discontinuation and Reduction)

Protocol paper

INTRODUCTION

Schizophrenia and related conditions affect up to 1% of the population (1), and are associated with long-term suffering and disability, premature death, physical illness and high costs to individuals and society (2). Recommended treatment for people with recurrent episodes consists of continuing antipsychotic medication,(3,4). Current guidelines do not recommend attempts at reduction or discontinuation of antipsychotics after the first episode, and in practice, antipsychotic treatment is often 'for life'. In spite of ongoing treatment, many people remain functionally impaired. In one study 25% of people with schizophrenia had severe social disabilities after 15 years, and only 14% had none (5).

The evidence base for long-term antipsychotic treatment consists of studies showing lower relapse rates with maintenance treatment compared with discontinuation (6). However, there are acknowledged methodological problems with these studies (7). In addition, several long-term non-randomised cohorts studies find worse outcomes in people who take continuous treatment compared to those who do not, although confounding by indication is likely to be relevant (8,9). Criticism of randomised controlled trials includes the fact that most focus on relapse and neglect other outcomes. Follow-up is generally short; only six of 65 studies included in a recent meta-analysis by Leucht et al (6) had followed the participants up for more than a year, and longer duration of follow-up was found to be associated with less difference between maintenance and discontinuation (6,10). Moreover, relapse rates may have been inflated by abrupt discontinuation and misidentification of withdrawal-related adverse effects (11,12). Some evidence suggests gradual discontinuation may reduce risk of relapse compared with abrupt discontinuation (13), although this difference was not confirmed in the analysis by Leucht et al (6). However, the average taper of 28 days for gradual medication withdrawal may not have prevented discontinuation-related effects in those people who had been taking antipsychotics for many years.

Long-term antipsychotic medication is associated with potentially serious physical complications, including diabetes, tardive dyskinesia, and cardiovascular disease (14–17).

For peer review only - http://bmjopen.bmj.com/site/about/guidelines.xhtml

Other adverse effects, such as sexual dysfunction, sedation, emotional blunting and akathisia may be debilitating and unpleasant (18–20). Previous research has found that many patients find the adverse effects of antipsychotics burdensome, and would like to try and discontinue the treatment at some stage(21–23).

There are few long-term follow-ups of people from randomised studies of antipsychotic discontinuation. A 7-year follow-up of an 18 month open trial conducted with people with first episode psychosis found that assignment to a gradual antipsychotic reduction programme was associated with better rates of social recovery and equal rates of relapse compared to maintenance treatment (24). Improved levels of neurocognitive performance exhibited by those randomised to antipsychotic reduction at 18 months suggest a possible mechanism for the long-term differences in functional outcome (25). In contrast, recent data from a 10-year follow-up of a one-year, placebo-controlled trial of quetiapine in people with first-episode psychosis reported higher rates of a composite 'poor outcome' in people originally randomised to placebo. There were no differences, however, in overall symptom measures, social functioning or quality of life (26).

Therefore, although continuing antipsychotic treatment has become the norm, it remains unclear whether it has an optimal risk-benefit balance for all people with psychosis or schizophrenia. In particular, more evidence on the effects of gradual reduction of antipsychotics on short and longer term outcomes could inform practice in this area. Effects on social functioning are particularly important to study because of evidence that long-term antipsychotic treatment may impair social functioning, despite improving symptoms or reducing relapse in the short-term (24; 27).

Further studies are currently being conducted in the first-episode psychosis population (28). To date, however, there is no study using gradual and flexible reduction with long-term follow-up in people with more than one episode.

Aims of trial

The current trial was designed to compare the benefits and harms of a gradual programme of dose reduction and discontinuation of antipsychotic treatment, under clinician guidance, with maintenance treatment in people with a diagnosis of schizophrenia or a related disorder. In particular, the study was set up to test whether such a strategy can improve functional outcomes

For peer review only - http://bmjopen.bmj.com/site/about/guidelines.xhtml

in people with recurrent or chronic psychosis while minimising the risk of worsening symptoms or relapse.

METHODS AND ANALYSIS

Study design

The trial consists of an open, parallel group randomised trial with concealed, individual randomisation. Randomisation is conducted through an independent, internet-based system linked with the database (https://www.sealedenvelope.com/randomisation/internet/) with 1:1 allocation. There are no replacements for participants who drop out or otherwise cannot comply with study procedures.

Participants and clinicians are aware of allocation, but outcome assessors are maintained blind to intervention arm as far as possible. This is achieved by nominating separate blinded and unblinded researchers at each site, and ensuring that blinded researchers are not exposed to discussions or written information that would reveal allocation. Following follow-up assessment blinded research staff record if they suspect they have guessed arm allocation.

The trial lasts for two years, with follow-up assessments conducted at 6, 12 and 24 months post randomisation. The trial design is presented in Figure 1. Ethical approval includes the possibility of conducting a longer-term follow-up, and resources will be sought to support this at a later date.

INSERT FIGURE 1 HERE

Interventions

The antipsychotic reduction protocol was developed after consultation with professionals, academics and the trial's RADAR Lived Experience Advisory Panel (LEAP). Both the antipsychotic reduction and maintenance protocols are administered by treating psychiatrists. For those participants randomised to the antipsychotic medication reduction arm, an individualised reduction schedule is devised by the research team for each participant based on clinical judgement and adjusted according to the participant's initial antipsychotic regimen. The dose is reduced incrementally every one or two months, focusing on one drug at a time

For peer review only - http://bmjopen.bmj.com/site/about/guidelines.xhtml

where participants are taking more than one antipsychotic. The rate of reduction varies according to baseline dose, with most schedules aiming for discontinuation within 12 months, but some lasting longer where baseline doses are high. Treating psychiatrists are asked to see the participants who have been randomised to antipsychotic reduction approximately every two months for the duration of the reduction, to adjust the medication regimen and monitor mental state. The participants are offered the option to discontinue antipsychotic medication completely if the reduction progresses well, or to reduce to a very low dose, defined as the equivalent of 2mg of haloperidol a day or less, which is lower than the minimum recommended therapeutic dose for most antipsychotics. This is twice the dose that was defined as a 'low dose' in the Dutch first episode study (24), since some research suggests that doses of antipsychotic medication in people with first episodes should be around half those recommended for people with more established disorders (29,30). Guidance on the antipsychotic reduction strategy stresses the need for flexibility, and includes a suggested protocol for the treatment of adverse reactions to withdrawal or symptom exacerbation. Participants randomised to maintenance treatment are requested not to make major reductions in their dose of antipsychotic medication during the trial period. Increases in dose are permitted within the protocol, as are changes to a different antipsychotic agent at the same equivalent dose and minor dose reductions to address side effects.

Participants are monitored by their care team according to usual practice. Patient records are scrutinised by members of the research team every two months to monitor the progress of the antipsychotic reduction and adherence to the maintenance protocol. Any deviations, such as participants allocated to reduction not starting or halting their reduction, are discussed with the treating clinician. All changes in antipsychotic doses and use of other medications is recorded throughout the study.

Other drug treatment and interventions such as psychological therapies may be used as indicated throughout the trial.

Inclusion and exclusion criteria

The eligibility criteria are detailed in Table 1. They are designed to make the trial as generalizable as possible to routine clinical practice, whilst minimising the risks of antipsychotic reduction in those with a history of posing a serious risk to self or others.

For peer review only - http://bmjopen.bmj.com/site/about/guidelines.xhtml

INSERT TABLE 1 HERE

Outcome measures

The primary outcome is social functioning at two-year follow-up. This will be measured using the Social Functioning Scale (SFS). The scale was developed in 1990, and has good reliability (31,32). It can demonstrate change over time (33–35) and distinguishes different types of antipsychotic treatment (33–36). It also distinguishes between people in remission and those with an ongoing episode (37), people with long versus short duration of untreated psychosis (DUP) (38), people who convert to psychosis versus those who do not in an 'ultra-high risk' cohort (34), and between people who are employed versus unemployed (31). This scale was preferred to the Groningen Social Disability Scale (GSDS) (39), which was used in the Dutch first-episode study, because of the GSDS is long, requires lengthy training and has a strong focus on relationships rather than functioning.

The main measure of the safety of the trial is severe relapse which is defined as admission to acute psychiatric in-patient treatment. This was selected because it is an objective criterion, can be precisely dated and can be obtained through patient records. Previous antipsychotic withdrawal studies have defined relapse in numerous different ways, with no consensus on defining criteria. Therefore, the current trial will also use an expert endpoint committee to assess the presence or absence of relapse based on blinded information from clinical case-notes, based on pre-defined criteria and guidance.

Other outcome measures include: symptoms as measured by the Positive and Negative Syndrome Scale (PANSS) (40), subjective quality of life as measured by the Manchester Short Assessment of quality of life (MANSA) (41), adverse effects of antipsychotics measured by a modified version of the Glasgow Antipsychotic Side-effect Scale (GASS) (42), body weight, sexual dysfunction as measured by the Arizona Sexual Experiences Scale (ASEX) (43), Work Productivity and Activity Impairment- General Health (WPAI) questionnaire (44), neuropsychological function (measured by a brief battery of tests designed for this trial, recovery as measured by the Questionnaire about the Process of Recovery (QPR) (45), ICECAP-A (quality of life), and EQ-5D-5L (quality of life).

For peer review only - http://bmjopen.bmj.com/site/about/guidelines.xhtml

In addition, process data will be collected, including dose of antipsychotic medication (in haloperidol equivalents), patient satisfaction as measured by the Client Satisfaction Questionnaire CSQ (46) and antipsychotic medication adherence as measured by Medication Adherence Rating Scale MARS-5 (47).

Table 2 shows the time points at which each measure will be completed. All outcome measures are administered by research staff who have received standardised training. Data collected during assessments is stored in a secure online data management system provided by the Sponsor. Participant retention is promoted by sending correspondence and study newsletters to participants facilitate engagement throughout the trial duration.

INSERT TABLE 2 HERE

Recruitment

Participants are recruited from a variety of clinical teams within mental health services across the United Kingdom. Potential participants are identified initially by clinical staff or recruited by advertisements placed in clinical settings. Those who agree are sent further information about the study and then a baseline assessment is arranged after further discussion. Assessments are conducted at the patient's home or in clinical premises according to patient preference.

Risk reduction

The eligibility criteria are designed to exclude people with known high risks of causing harm to themselves or other people. In addition, the gradual nature of the antipsychotic reduction will enable detection and treatment of early signs of relapse. All participants will receive usual care and monitoring of their mental state and behaviour by their clinical team. Those randomised to antipsychotic reduction will have increased contact with a psychiatrist for the duration of the reduction, mirroring usual clinical practice with someone undergoing a significant reduction of medication.

For peer review only - http://bmjopen.bmj.com/site/about/guidelines.xhtml

Qualitative assessment

There will be a qualitative sub-study involving around 20-30 participants from the antipsychotic reduction group and around 12 clinicians with experience of delivering the antipsychotic reduction programme. This study has two aims: to collect data on participant and clinician experience of trial processes, and to explore in detail experiences of antipsychotic reduction and discontinuation from the patient perspective. Participants will be identified towards the end of follow up, using purposive sampling to obtain variation in clinical profile, experience of reduction, completion of the antipsychotic reduction protocol and experience of relapse.

Participants will be interviewed using a semi-structured interview schedule developed in collaboration with the RADAR Lived Experience Advisory Panel (LEAP). This will explore patients' experiences of the intervention and its impact on their mental health and wider lives, the acceptability of antipsychotic reduction, satisfaction with available support, and responses to adverse events. Interviews will be conducted with early participants following the 24-month follow-up interviews so as not to confound the main trial results. Interviews with clinicians will explore their experiences of implementing different aspects of the antipsychotic reduction strategy, relations with the research team, acceptability of medication monitoring procedures, and responses to adverse events. The data will be analysed using thematic analysis.

Statistical analysis

The primary outcome (Social Functioning Scale, SFS) will be analysed using generalised mixed models, accounting for baseline and treatment periods. The principal analysis will be undertaken using the intention to treat population, including all available data, without imputation. Supportive analyses will include analysis of different time periods, analysis using repeated measurements (where all subjects with data at one or more post intervention assessment will be included), exploring potential centre effects and making pessimistic assumptions by group on the patient outcome (to assess the thresholds to the impact of 'missingness') because of the likely pattern of data missing not at random.

The proportions of patients who experience a 'severe relapse' (psychiatric hospital admission) will be compared between the randomised groups at two year follow-up, and survival analysis

For peer review only - http://bmjopen.bmj.com/site/about/guidelines.xhtml

using Cox constant proportional hazards models including centres as marginal frailty terms will also be performed. We will undertake supportive threshold analyses where any loss to follow up will be considered according to extreme data patterns – e.g. assuming all those who were lost in the antipsychotic withdrawal group had a relapse and all those in the maintenance group did not, and thus quantifying the extent of possible effects of missingness and thus the robustness of the main result.

A full statistical analysis plan will be completed and agreed prior to any analysis or unblinding of the data, including planned subgroup analyses and with detailed description of the statistical processes to be used.

Economic analysis

The economic evaluation will evaluate the cost-effectiveness of the antipsychotic reduction strategy compared to maintenance treatment over 24 months. The principal analysis will be the incremental cost per quality adjusted life year gained from the health and social services cost perspective using the EQ-5D-5L to calculate quality adjusted life years (QALYs) as recommended by NICE (48). A secondary analysis will include the cost per capability adjusted life year (CALY) gained of the antipsychotic reduction strategy compared to maintenance treatment over 24 months using the ICECAP-A, argued to have better face validity (49). Additional sensitivity analyses will explore the cost-effectiveness of antipsychotic reduction compared to maintenance capturing the QoL loss associated with antipsychotics side effects using specific disutility values for antipsychotic side effects from the published literature (50).

The Work Productivity and Activity Impairment- General Health (WPAI) questionnaire and associated formula (44) will be used to give a monetary value to impact on employment. Health and social care data costs using published sources will be used to calculate costs for the primary health and social care analysis. A secondary analysis will be conducted from a societal perspective to capture the impact on employment, criminal justice, benefits, family and close others

All costs and outcomes will be discounted in line with NICE guidance at 3.5% per year. Bootstrapping will be used to construct confidence intervals for total mean costs, QALYs and CALYs and to construct cost effectiveness acceptability curves and cost-effectiveness planes. Missing data will be handled in the same way as stated by the statistical analysis plan.

For peer review only - http://bmjopen.bmj.com/site/about/guidelines.xhtml

Sample size calculation

The primary outcome is social functioning, measured using the Social Functioning Scale (31). The literature suggests a minimum difference of between four and five points would be clinically significant, since a difference of four to five points appears to differentiate between patients with good and poor outcomes (37,51,52).

Using a conventional α of 5% (two sided) and taking a SD of 8.8 derived from the literature (53), a sample size of 134 evaluable participants is required to provide 90% power to identify a five point difference, and a sample of 206 is required to identify a four point difference.

The trial also aimed to establish the safety of the antipsychotic reduction strategy by being powered to detect differences in rates of 'severe' relapse (psychiatric hospitalisation), the principal safety measure. We believe that an increased risk of up to 10% would be acceptable to many clinicians and patients if it is balanced by the important outcomes of an improvement in social functioning and reduced side effects. We derived event rates for hospitalisation from those reported in the Leucht et al (6) meta-analysis of antipsychotic discontinuation studies.

We conducted a non-inferiority calculation using a 10% margin of difference. Using a non-inferiority boundary of 10% event rates (severe relapse), with an alpha of 0.05, requires a sample size of 372 for 90% power to exclude a difference of 10% between groups. Adding 15% for attrition brings this up to 402. The lower confidence interval on the absolute scale would exclude a difference of 10% in the situation where non inferiority was achieved. 402 participants provides 90% power to detect a difference of 3.1 points on the primary efficacy outcome (the SFS), including an allowance of 15% for attrition or unexpected challenges to the assumptions.

It is acknowledged that the non-inferiority approach may be unhelpful in these circumstances, as different people may have substantially different views on what is a reasonable non-inferiority boundary. Further, the assumptions required on issues such as the event rate for the non-inferiority assumption are strong, and modest changes in rates can have a substantial impact on the statistical power of the study making the proposed non inferiority boundary unhelpful in certain circumstances. Thus the principal aim of the study with regards to severe relapse is to provide a robust estimate of the event rates in each randomised condition, and the

For peer review only - http://bmjopen.bmj.com/site/about/guidelines.xhtml

difference between them, describing measurement error with appropriate confidence intervals in order to allow individual patients and their clinicians to make an informed decision on treatment options.

PATIENT AND PUBLIC INVOLVEMENT

Testing this research question is a response to a long-standing demand from the service user and carer community for an alternative to long-term antipsychotic medication. Many service users and carers are extremely concerned about the possible long-term side effects of antipsychotic medication. The study has four co-applicants with a patient or public perspective who attend project management meetings; a carer, a service user, an experienced coordinator and researcher, and a staff member of a relevant charity. There is also a LEAP (Lived Experience Advisory Panel), made up of people with expertise in antipsychotic medication through personal use or as a carer for someone with psychosis. It meets regularly to discuss the progress of the study and to contribute to its development, for example, advising on the antipsychotic reduction strategy. The LEAP will be particularly involved in the qualitative study as part of the process evaluation. They will also be involved in providing feedback on study outputs including a regular newsletter and disseminating research results. Results of the study will be disseminated to participants at their request.

MONITORING

The trial is monitored by a representative of the Sponsor according to the agreed trial-specific monitoring plan. A Data Safety and Monitoring Board (DSMB) and Programme Steering Committee provide independent oversight of the trial. The DSMB safeguards the interests of trial participants by assessing the safety and efficacy of the interventions during the trial, and monitoring its conduct and it makes recommendations to the steering committee. There will be no formal interim analysis, but the DSMB will continually review all adverse events data. The trial will be stopped if it is judged that there is a substantial increase in serious adverse events that are likely to be related to the intervention.

SAFETY & ADVERSE EVENTS

All adverse events are recorded with a description of the event, and are assessed for severity, causality, expectedness and seriousness.

For peer review only - http://bmjopen.bmj.com/site/about/guidelines.xhtml

ETHICS AND DISSEMINATION

The trial received approval from the Brent Ethics committee on 27/10/2016. The Research Ethics Committee (REC) reference is 16/LO/1507. The trial was registered with the International Standard Randomised Controlled Trials register on 7/2/2017, registration number: ISRCTN90298520 and with ClinicalTrials.gov on 15/6/2018. All trial data is handled according to the UK Data Protection Act 1998. Substantial protocol amendments are documented and submitted for ethical and regulatory approval prior to implementation.

Written informed consent is obtained prior to participation in the trial, following a full explanation of the aims, methods, anticipated benefits and hazards of the trial. A formal assessment of each participant's capacity to provide consent is completed first. Consent is an ongoing process and researchers ask participants for verbal consent at each follow-up time-point.

Results will be published in peer-reviewed journals and disseminated to the public and the media. All contributors must meet the 'Authorship Criteria', as recommended by The International Committee of Medical Journal Editors.

Current trial status

Recruitment started in March 2016 and will continue until 2020.

DISCUSSION

Better evidence is required about the balance of risks and benefits of a supported programme of antipsychotic reduction and discontinuation under the guidance of a clinician for the many people who have a diagnosis of schizophrenia or recurrent psychoses. The current study will be the first study conducted with people with more than one psychotic episode to employ a gradual and supported method of antipsychotic reduction, to look at a wide range of outcomes giving priority to social functioning, and to follow people up for a reasonable duration. Further

For peer review only - http://bmjopen.bmj.com/site/about/guidelines.xhtml

follow-ups are envisaged after the official end of the trial in order to provide data on longer-term outcomes.

The Dutch first-episode study suggested that long-term outcomes after a period of antipsychotic dose reduction differ from short-term outcomes (24,54). Although relapse rates are increased initially compared with maintenance treatment, over time these equalise. Social functioning, which was not affected in the short-term, was considerably improved by the time of the seven-year follow-up assessment. Social functioning is a measure of independence and personal efficacy. If the current trial results in improvements in social functioning this could reduce an individual's reliance on services and produce significant economic benefits. From the individual's point of view, better social functioning reflects a more fulfilling and socially integrated life. A strategy that can successfully reduce the use of antipsychotic medication is also likely to be associated with health benefits and improvements in quality of life secondary to the reduction of adverse effects.

The outcomes of the current trial will provide good evidence to inform patients and clinicians about the likely consequences of reducing and discontinuing long-term antipsychotic treatment in a gradual manner in a clinical setting. Many patients currently want to consider this option, but existing data on the range of relevant outcomes is limited. Providing further information will facilitate a more collaborative approach to long-term antipsychotic treatment.

For peer review only - http://bmjopen.bmj.com/site/about/guidelines.xhtml

For peer review only

PATIENT CONSENT FOR PUBLICATION: Not required

ACKNOWLEDGEMENTS

This research is sponsored by Priment Clinical Trials Unit, University College London (Sponsor's reference number: 15/0947) and the authors would like to thank members of Priment for their support for the trial. The RADAR research team also acknowledges the support of the National Institute for Health Research Clinical Research Network and North East London NHS Foundation Trust. The research team thank the RADAR Lived Experience Advisory Panel (LEAP) for their expertise, guidance and support. With thanks also to the independent members of the Programme Steering Committee and the Data and Safety Monitoring Board.

AUTHORS' CONTRIBUTIONS

JM developed the original concept for the trial and drafted the protocol. All authors contributed to the trial design and methodology. JM, GL, NF, SJ, TB, NM, VP, RH, LK, RS, KD, RH & SP are grantholders. NF provided statistical expertise. NC and RC provided trial management and updated the protocol. JM drafted the manuscript, and JM and NC edited subsequent drafts. All authors reviewed and commented on drafts of the paper and approved the final manuscript version. The study Sponsor has contributed to the study design, data collection, management, analysis and interpretation of data.

FUNDING

This report is independent research funded by the National Institute for Health Research (Programme Grants for Applied Research, Research into Antipsychotic Discontinuation And Reduction (RADAR), RP-PG-0514-20004). The views expressed in this publication are those of the author(s) and not necessarily those of the NHS, the National Institute for Health Research or the Department of Health. The NIHR will not have any role during the study execution, analyses, or interpretation of data.

For peer review only - http://bmjopen.bmj.com/site/about/guidelines.xhtml

For peer review only

COMPETING INTERESTS

TREB: In the last three years, TREB has been a member of scientific advisory boards for Otsuka/Lundbeck, Newron Pharmaceuticals and Gedeon Richter and received speaker fees from Janssen.

GL has acted as an expert witness in cases concerning litigation about antidepressants.

No other authors have competing interests to declare.

For peer review only

For peer review only - http://bmjopen.bmj.com/site/about/guidelines.xhtml

REFERENCES

1. McGrath J, Saha S, Chant D, Welham J. Schizophrenia: A Concise Overview of Incidence, Prevalence, and Mortality. *Epidemiol Rev* 2008;14:30(1):67–76. https://doi.org/10.1093/epirev/mxn001

2. McCrone P, Dhanasiri S, Patel A, Knapp.M., Lawton S. Paying the price. the cost of mental health care in England to 2026. London: Kings Fund; 2008.

3. Hasan A, Falkai P, Wobrock T, Lieberman J, Glenthoj B, Gattaz WF, et al. World Federation of Societies of Biological Psychiatry (WFSBP) Guidelines for Biological Treatment of Schizophrenia, Part 2: Update 2012 on the long-term treatment of schizophrenia and management of antipsychotic-induced side effects. *World J Biol Psychiatry* 2013;14(1):2–44. https://doi.org/10.3109/15622975.2012.739708

4. National Institute for Health and Care Excellence. Psychosis and schizophrenia in adults: treatment and management. London: NICE; 2014.

5. Wiersma D, Wanderling J, Dragomirecka E, Ganev K, Harrison G, an der HW, et al. Social disability in schizophrenia: its development and prediction over 15 years in incidence cohorts in six European centres. *Psychol Med* 2000;30(5):1155-67. https://doi.org/10.1017/S0033291799002627

6. Leucht S, Tardy M, Komossa K, Heres S, Kissling W, Davis JM. Maintenance treatment with antipsychotic drugs for schizophrenia. *Cochrane Database Syst Rev* 2012;5:CD008016. https://doi.org/10.1002/14651858.CD008016.pub2

7. Leucht S, Heres S, Hamann J, Kane JM. Methodological Issues in Current Antipsychotic Drug Trials. *Schizophr Bull* 2008;9;34(2):275–85. https://doi.org/10.1093/schbul/sbm159

8. Harrow M, Jobe TH, Faull RN. Do all schizophrenia patients need antipsychotic treatment continuously throughout their lifetime? A 20-year longitudinal study. *Psychol Med* 2012;42(10):2145–55. https://doi.org/10.1017/S0033291712000220

9. Moilanen JM, Haapea M, Jääskeläinen E, Veijola JM, Isohanni MK, Koponen HJ, et al. Long-term antipsychotic use and its association with outcomes in schizophrenia – the Northern Finland Birth Cohort 1966. *Eur Psychiatry* 2016;36:7–14. https://doi.org/10.1016/j.eurpsy.2016.03.002

10. Gilbert PL, Harris MJ, McAdams LA, Jeste DV. Neuroleptic withdrawal in schizophrenic patients. A review of the literature. *Arch Gen Psychiatry* 1995;52(3):173-88. https://doi.org/10.1001/archpsyc.1995.03950150005001

11. Baldessarini RJ, Viguera AC. Neuroleptic withdrawal in schizophrenic patients. *Arch Gen Psychiatry* 1995;52(3):189-92. https://doi.org/10.1001/archpsyc.1995.03950150021002

12. Whitaker R. Anatomy of an Epidemic. New York: Crown Publishers; 2010.

13. Viguera AC, Baldessarini RJ, Hegarty JD, van Kammen DP, Tohen M. Clinical risk following abrupt and gradual withdrawal of maintenance neuroleptic treatment. *Arch Gen Psychiatry* 1997;54(1):49–55. https://doi.org/10.1001/archpsyc.1997.01830130055011

For peer review only - http://bmjopen.bmj.com/site/about/guidelines.xhtml

14. Osborn DPJ, Levy G, Nazareth I, Petersen I, Islam A, King MB. Relative Risk of Cardiovascular and Cancer Mortality in People With Severe Mental Illness From the United Kingdom's General Practice Research Database. *Arch Gen Psychiatry* 2007;64(2):242-9. https://doi.org/10.1001/archpsyc.64.2.242

15. Ray WA, Chung CP, Murray KT, Hall K, Stein CM. Atypical antipsychotic drugs and the risk of sudden cardiac death. *N Engl J Med* 2009;15;360(3):225-35. https://doi.org/10.1056/NEJMoa0806994

16. Yood MU, deLorenze G, Quesenberry CP, Oliveria SA, Tsai A-L, Willey VJ, et al. The incidence of diabetes in atypical antipsychotic users differs according to agent-results from a multisite epidemiologic study. *Pharmacoepidemiol Drug Saf* 2009;18(9):791–9. https://doi.org/10.1002/pds.1781

17. Correll CU, Schenk EM. Tardive dyskinesia and new antipsychotics. *Curr Opin Psychiatry* 2008;21(2):151-6. https://doi.org/10.1097/YCO.0b013e3282f53132

18. Moncrieff J, Cohen D, Mason JP. The subjective experience of taking antipsychotic medication: a content analysis of Internet data. *Acta Psychiatr Scand* 2009;120(2):102–11. https://doi.org/10.1111/j.1600-0447.2009.01356.x

19. Barbui C, Nose M, Bindman J, Schene A, Becker T, Mazzi MA, et al. Sex Differences in the Subjective Tolerability of Antipsychotic Drugs. *J Clin Psychopharmacol* 2005;25(6):521–6. https://doi.org/10.1097/01.jcp.0000185423.15891.02

20. Hatano M, Kamei H, Kato A, Takeuchi I, Hanya M, Uno J, et al. Assessment of the Latent Adverse Events of Antipsychotic Treatment Using a Subjective Questionnaire in Japanese Patients with Schizophrenia. *Clin Psychopharmacol Neurosci* 2017;31;15(2):132–7. https://doi.org/10.9758/cpn.2017.15.2.132

21. Williams K, Pinfold V. Side Effects: Mental health service users experiences of the side effects of antipsychotic medication. London: Rethink; 2006.

22. Read J. Psychiatric Drugs: Key issues and service user perspectives. Basingstoke, Hants: Palgrave Macmillan; 2009.

23. Morant N, Azam K, Johnson S, Moncrieff J. The least worst option: user experiences of antipsychotic medication and lack of involvement in medication decisions in a UK community sample. *J Ment Health* 2018;4;27(4):322–8. https://doi.org/10.1080/09638237.2017.1370637

24. Wunderink L, Nieboer RM, Wiersma D, Sytema S, Nienhuis FJ. Recovery in Remitted First-Episode Psychosis at 7 Years of Follow-up of an Early Dose Reduction/Discontinuation or Maintenance Treatment Strategy: Long-term Follow-up of a 2-Year Randomized Clinical Trial. *JAMA Psychiatry* 2013;1;70(9):913. https://doi.org/10.1001/jamapsychiatry.2013.19

25. Faber G, Smid HGOM, Van Gool AR, Wunderink L, Wiersma D, van den Bosch RJ. Neurocognition and recovery in first episode psychosis. *Psychiatry Res* 2011;188(1):1–6. https://doi.org/10.1016/j.psychres.2010.11.010

26. Hui CLM, Honer WG, Lee EHM, Chang WC, Chan SKW, Chen ESM, et al. Long-term effects of discontinuation from antipsychotic maintenance following first-episode schizophrenia and related disorders: a 10 year follow-up of a randomised, double-blind trial. *Lancet Psychiatry* 2018;5(5):432–42. https://doi.org/10.1016/S2215-0366(18)30090-7

For peer review only - http://bmjopen.bmj.com/site/about/guidelines.xhtml

27. Wils RS, Gotfredsen DR, Hjorthøj C, Austin SF, Albert N, Secher RG, et al. Antipsychotic medication and remission of psychotic symptoms 10 years after a first-episode psychosis. *Schizophr Res* 2017;182:42-48. doi: 10.1016/j.schres.2016.10.030.

28. Stürup AE, Jensen HD, Dolmer S, Birk M, Albert N, Nielsen M, et al. TAILOR – tapered discontinuation versus maintenance therapy of antipsychotic medication in patients with newly diagnosed schizophrenia or persistent delusional disorder in remission of psychotic symptoms: study protocol for a randomized clinical trial. *Trials* [Internet]. 2017 Dec [cited 2019 Feb 18];18(1). Available from: http://trialsjournal.biomedcentral.com/articles/10.1186/s13063-017-2172-4

29. McEvoy JP, Hogarty GE, Steingard S. Optimal dose of neuroleptic in acute schizophrenia. A controlled study of the neuroleptic threshold and higher haloperidol dose. *Arch Gen Psychiatry* 1991;48(8):739–45. https://doi.org/10.1001/archpsyc.1991.01810320063009

30. Oosthuizen P, Emsley RA, Turner J, Keyter N. Determining the optimal dose of haloperidol in first-episode psychosis. *J Psychopharmacol (Oxf)* 2001;15(4):251–5. https://doi.org/10.1177/026988110101500403

31. Birchwood M, Smith J, Cochrane R, Wetton S, Copestake S. The Social Functioning Scale. The Development and Validation of a New Scale of Social Adjustment for use in Family Intervention Programmes with Schizophrenic Patients. *Br J Psychiatry* 1990;157(06):853–9. https://doi.org/10.1192/bjp.157.6.853

32. Vázquez Morejón AJ, Jiménez Ga-Bóveda R. Social Functioning Scale: new contributions concerning its psychometric characteristics in a Spanish adaptation. *Psychiatry Res* 2000;93(3):247–56. https://doi.org/10.1016/S0165-1781(99)00123-7

33. Ciudad A, Olivares JM, Bousoño M, Gómez JC, Álvarez E. Improvement in social functioning in outpatients with schizophrenia with prominent negative symptoms treated with olanzapine or risperidone in a 1 year randomized, open-label trial. *Prog Neuropsychopharmacol Biol Psychiatry* 2006;30(8):1515–22. https://doi.org/10.1016/j.pnpbp.2006.05.010

34. Jang JH, Shin NY, Shim G, Park HY, Kim E, Jang G-E, et al. Longitudinal Patterns of Social Functioning and Conversion to Psychosis in Subjects at Ultra-High Risk. *Aust N Z J Psychiatry* 2011;45(9):763–70. https://doi.org/10.3109/00048674.2011.595684

35. Sheridan AJ, Drennan J, Coughlan B, O'Keeffe D, Frazer K, Kemple M, et al. Improving social functioning and reducing social isolation and loneliness among people with enduring mental illness: Report of a randomised controlled trial of supported socialisation. *Int J Soc Psychiatry* 2015;61(3):241–50. https://doi.org/10.1177/0020764014540150

36. Koshikawa Y, Takekita Y, Kato M, Sakai S, Onohara A, Sunada N, et al. The Comparative Effects of Risperidone Long-Acting Injection and Paliperidone Palmitate on Social Functioning in Schizophrenia: A 6-Month, Open-Label, Randomized Controlled Pilot Trial. *Neuropsychobiology* 2016;73(1):35–42. https://doi.org/10.1159/000442209

37. Jaracz K, Górna K, Kiejda J, Grabowska-Fudala B, Jaracz J, Suwalska A, et al. Psychosocial functioning in relation to symptomatic remission: A longitudinal study of first episode schizophrenia. *Eur Psychiatry* 2015;30(8):907–13. https://doi.org/10.1016/j.eurpsy.2015.08.001

For peer review only - http://bmjopen.bmj.com/site/about/guidelines.xhtml

38. Barnes TRE, Leeson VC, Mutsatsa SH, Watt HC, Hutton SB, Joyce EM. Duration of untreated psychosis and social function: 1-year follow-up study of first-episode schizophrenia. *Br J Psychiatry* 2008;193(03):203–9. https://doi.org/10.1192/bjp.bp.108.049718

39. Wiersma D, DeJong A, Ormel J. The Groningen Social Disabilities Schedule: development, relationship with I.C.I.D.H., and psychometric properties. *Int J Rehabil Res* 1988;11(3):213-24.

40. Kay SR, Fiszbein A, Opler LA. The Positive and Negative Syndrome Scale (PANSS) for Schizophrenia. *Schizophr Bull* 1987;1;13(2):261–76. https://doi.org/10.1093/schbul/13.2.261

41. Priebe S, Huxley P, Knight S, Evans S. Application and Results of the Manchester Short Assessment of Quality of Life (Mansa). *Int J Soc Psychiatry* 1999;45(1):7–12. https://doi.org/10.1177/002076409904500102

42. Waddell L, Taylor M. A new self-rating scale for detecting atypical or second-generation antipsychotic side effects. *J Psychopharmacol (Oxf)* 2008;22(3):238–43. https://doi.org/10.1177/0269881107087976

43. McGahuey CA, Gelenberg AJ, Laukes CA, Moreno FA, Delgado PL, McKnight KM, et al. The Arizona Sexual Experience Scale (ASEX): reliability and validity. *J Sex Marital Ther* 2000 Jan;26(1):25-40. https://doi.org/10.1080/009262300278623

44. Reilly MC, Zbrozek AS, Dukes EM. The validity and reproducibility of a work productivity and activity impairment instrument. *Pharmacoeconomics* 1993;4(5):353–65. https://doi.org/10.2165/00019053-199304050-00006

45. Law H, Neil ST, Dunn G, Morrison AP. Psychometric properties of the Questionnaire about the Process of Recovery (QPR). *Schizophr Res* 2014;156(2–3):184–9. https://doi.org/10.1016/j.schres.2014.04.011

46. Atkisson CC, Zwick R. The Client Satisfaction Questionnaire: Psychometric properties and correlations with service utilisation and psychotherapy outcome. *Evaluation and Program Planning* 1982;5(3):233-7. https://doi.org/10.1016/0149-7189(82)90074-X

47. Mahler C, Hermann K, Horne R, Ludt S, Haefeli WE, Szecsenyi J, et al. Assessing reported adherence to pharmacological treatment recommendations. Translation and evaluation of the Medication Adherence Report Scale (MARS) in Germany. *J Eval Clin Pract* 2010;16(3):574-9. https://doi.org/10.1111/j.1365-2753.2009.01169.x

48. EuroQol Group. EuroQol--a new facility for the measurement of health-related quality of life. *Health Policy* 1990;16(3):199-208. https://doi.org/10.1016/0168-8510(90)90421-9

49. Brazier J, Connell J, Papaioannou D, Mukuria C, Mulhern B, Peasgood T, et al. A systematic review, psychometric analysis and qualitative assessment of generic preference-based measures of health in mental health populations and the estimation of mapping functions from widely used specific measures. *Health Technol Assess* 2014;18(34):vii-xxv,1. https://doi.org/10.3310/hta18340

50. Briggs A, Wild D, Lees M, Reaney M, Dursun S, Parry D, et al. Impact of schizophrenia and schizophrenia treatment-related adverse events on quality of life: direct utility elicitation. *Health Qual Life Outcomes* 2008;6(1):105. https://doi.org/10.1186/1477-7525-6-105

For peer review only - http://bmjopen.bmj.com/site/about/guidelines.xhtml

51. Hellvin T, Sundet K, Vaskinn A, Simonsen C, Ueland T, Andreassen OA, et al. Validation of the Norwegian version of the Social Functioning Scale (SFS) for schizophrenia and bipolar disorder: Validation of the SFS (Norwegian version). *Scand J Psychol* 2010;51(6):525–33. https://doi.org/10.1111/j.1467-9450.2010.00839.x

52. Leeson VC, Harrison I, Ron MA, Barnes TRE, Joyce EM. The Effect of Cannabis Use and Cognitive Reserve on Age at Onset and Psychosis Outcomes in First-Episode Schizophrenia. *Schizophr Bull* 2012;1;38(4):873–80. https://doi.org/10.1093/schbul/sbq153

53. Slupczynska-Kossobudzka E, Boguszewska L. Effects of Community Mobile Team Intervention in the Drewnica Hospital Catchment Area. 1. Patient Outcome. *Int J Soc Psychiatry* 1999;45(3):207–15. https://doi.org/10.1177/002076409904500308

54. Wunderink L, Nienhuis FJ, Sytema S, Slooff CJ, Knegtering R, Wiersma D. Guided discontinuation versus maintenance treatment in remitted first-episode psychosis: relapse rates and functional outcome. *J Clin Psychiatry* 2007;68(5):654-61. https://doi.org/10.4088/JCP.v68n0502

For peer review only - http://bmjopen.bmj.com/site/about/guidelines.xhtml

Table 1. Eligibility criteria

**Inclusion criteria**	
1.	Aged 18 years or older
2.	A clinical and/or ICD10 diagnosis of schizophrenia, schizoaffective disorder, delusional disorder or other non-affective psychosis
3.	More than one previous episode or psychotic exacerbation, or a single episode lasting more than one year
4.	Prescribed continuing antipsychotic medication
**Exclusion criteria**	
1.	Lack of capacity to consent to the trial.
2.	Insufficient command of spoken English to understand trial procedures
3.	Subject to a section of the Mental Health Act (MHA) that includes a requirement to take antipsychotic medication
4.	Clinician considers there will be a serious risk of harm to self or others
5.	Admitted to hospital or treated by a Home Treatment or Crisis Team within the last month
6.	Women who have a confirmed pregnancy
7.	Women who are breast-feeding
8.	Involvement in another Investigational Medicinal Product (IMP) trial

For peer review only - http://bmjopen.bmj.com/site/about/guidelines.xhtml

Table 2. Time points at which different outcomes will be assessed

*A = Demographic information (selected sections including weight and use of illicit drugs and alcohol); B = Diagnosis (established from clinical records); C = Social Functioning Scale (SFS); D = Positive and Negative Syndrome Scale (PANSS); E = Glasgow Antipsychotic Side-effect Scale (GASS); F = Client Satisfaction Questionnaire (CSQ-8); G = Manchester Short Assessment of quality of life (MANSA); H = Neuropsychological function tests; I = Medication Adherence Rating Scale (MARS-5); J = Relapse questionnaire ; K = Serious Adverse Events; L = EQ-5D-5L; M = ICECAP-A; N = Client Service Receipt Inventory ; O = Work Productivity and Activity Questionnaire; P = Schedule for economic data from patient records; Q = Questionnaire about the Process of Recovery (QPR); R = Arizona Sexual Experiences Scale (ASEX).

	**Baseline**			**Follow-up**		
**Visit Number**	**1**	**2 Pilot trial 3-month data collection**	**3 6-month follow-up**	**4 12-month follow-up**	**5 24-month follow-up**	**6 Qualitative evaluation**
**Informed Consent**	X					X
**Eligibility determination**	X					
**Protocol Assessments**	A-I, L-O, Q, R	K,	C-G, I-O, Q, R	C-O,Q, R	C- R	Indicative topic guide with sample of participants and psychiatrists
**Randomisation**	X					
**IMP administration**	X	X	X	X	X	
**Adverse Events review**	X	X	X	X	X	
**Medical notes review for prescribing information and fidelity to intervention protocols**	X	X	X	X	X	
**Concomitant Medication review**	X	X	X	X	X	

For peer review only - http://bmjopen.bmj.com/site/about/guidelines.xhtml

Figure 1. Trial Flow Chart

For peer review only

For peer review only - http://bmjopen.bmj.com/site/about/guidelines.xhtml

Figure 1. Trial Flow Chart

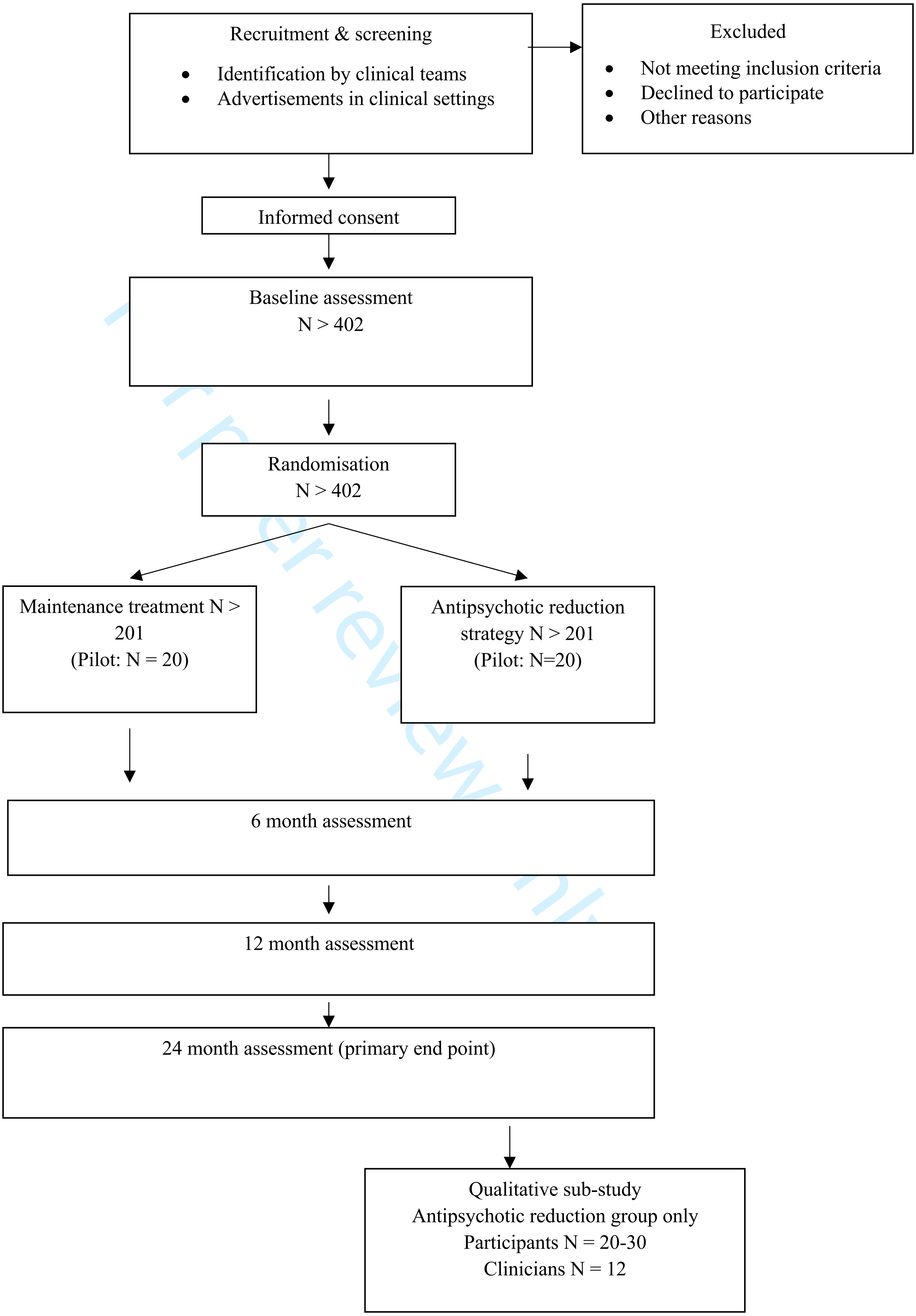

SPIRIT
STANDARD PROTOCOL ITEMS: RECOMMENDATIONS FOR INTERVENTIONAL TRIALS

SPIRIT 2013 Checklist: Recommended items to address in a clinical trial protocol and related documents*

Section/item	Item No	Description	Addressed on page number
**Administrative information**			
Title	1	Descriptive title identifying the study design, population, interventions, and, if applicable, trial acronym	1
Trial registration	2a	Trial identifier and registry name. If not yet registered, name of intended registry	2
	2b	All items from the World Health Organization Trial Registration Data Set	
Protocol version	3	Date and version identifier	
Funding	4	Sources and types of financial, material, and other support	16
Roles and responsibilities	5a	Names, affiliations, and roles of protocol contributors	1/16
	5b	Name and contact information for the trial sponsor	18
	5c	Role of study sponsor and funders, if any, in study design; collection, management, analysis, and interpretation of data; writing of the report; and the decision to submit the report for publication, including whether they will have ultimate authority over any of these activities	17
	5d	Composition, roles, and responsibilities of the coordinating centre, steering committee, endpoint adjudication committee, data management team, and other individuals or groups overseeing the trial, if applicable (see Item 21a for data monitoring committee)	18

For peer review only

Introduction

Background and rationale	6a	Description of research question and justification for undertaking the trial, including summary of relevant studies (published and unpublished) examining benefits and harms for each intervention	4/5
	6b	Explanation for choice of comparators	4/5
Objectives	7	Specific objectives or hypotheses	5
Trial design	8	Description of trial design including type of trial (eg, parallel group, crossover, factorial, single group), allocation ratio, and framework (eg, superiority, equivalence, noninferiority, exploratory)	6/12

Methods: Participants, interventions, and outcomes

Study setting	9	Description of study settings (eg, community clinic, academic hospital) and list of countries where data will be collected. Reference to where list of study sites can be obtained	7
Eligibility criteria	10	Inclusion and exclusion criteria for participants. If applicable, eligibility criteria for study centres and individuals who will perform the interventions (eg, surgeons, psychotherapists)	7/8
Interventions	11a	Interventions for each group with sufficient detail to allow replication, including how and when they will be administered	6/7
	11b	Criteria for discontinuing or modifying allocated interventions for a given trial participant (eg, drug dose change in response to harms, participant request, or improving/worsening disease)	6/7
	11c	Strategies to improve adherence to intervention protocols, and any procedures for monitoring adherence (eg, drug tablet return, laboratory tests)	6/7
	11d	Relevant concomitant care and interventions that are permitted or prohibited during the trial	6/7
Outcomes	12	Primary, secondary, and other outcomes, including the specific measurement variable (eg, systolic blood pressure), analysis metric (eg, change from baseline, final value, time to event), method of aggregation (eg, median, proportion), and time point for each outcome. Explanation of the clinical relevance of chosen efficacy and harm outcomes is strongly recommended	8/9
Participant timeline	13	Time schedule of enrolment, interventions (including any run-ins and washouts), assessments, and visits for participants. A schematic diagram is highly recommended (see Figure)	6

For peer review only - http://bmjopen.bmj.com/site/about/guidelines.xhtml

Sample size	14	Estimated number of participants needed to achieve study objectives and how it was determined, including clinical and statistical assumptions supporting any sample size calculations	12/13
Recruitment	15	Strategies for achieving adequate participant enrolment to reach target sample size	11

Methods: Assignment of interventions (for controlled trials)

Allocation:			
Sequence generation	16a	Method of generating the allocation sequence (eg, computer-generated random numbers), and list of any factors for stratification. To reduce predictability of a random sequence, details of any planned restriction (eg, blocking) should be provided in a separate document that is unavailable to those who enrol participants or assign interventions	6
Allocation concealment mechanism	16b	Mechanism of implementing the allocation sequence (eg, central telephone; sequentially numbered, opaque, sealed envelopes), describing any steps to conceal the sequence until interventions are assigned	6
Implementation	16c	Who will generate the allocation sequence, who will enrol participants, and who will assign participants to interventions	6
Blinding (masking)	17a	Who will be blinded after assignment to interventions (eg, trial participants, care providers, outcome assessors, data analysts), and how	6
	17b	If blinded, circumstances under which unblinding is permissible, and procedure for revealing a participant's allocated intervention during the trial	Na

Methods: Data collection, management, and analysis

Data collection methods	18a	Plans for assessment and collection of outcome, baseline, and other trial data, including any related processes to promote data quality (eg, duplicate measurements, training of assessors) and a description of study instruments (eg, questionnaires, laboratory tests) along with their reliability and validity, if known. Reference to where data collection forms can be found, if not in the protocol	9
	18b	Plans to promote participant retention and complete follow-up, including list of any outcome data to be collected for participants who discontinue or deviate from intervention protocols	9

Data management	19	Plans for data entry, coding, security, and storage, including any related processes to promote data quality (eg, double data entry; range checks for data values). Reference to where details of data management procedures can be found, if not in the protocol	9
Statistical methods	20a	Statistical methods for analysing primary and secondary outcomes. Reference to where other details of the statistical analysis plan can be found, if not in the protocol	12/13/14
	20b	Methods for any additional analyses (eg, subgroup and adjusted analyses)	12/13/14
	20c	Definition of analysis population relating to protocol non-adherence (eg, as randomised analysis), and any statistical methods to handle missing data (eg, multiple imputation)	12/13/14
**Methods: Monitoring**			
Data monitoring	21a	Composition of data monitoring committee (DMC); summary of its role and reporting structure; statement of whether it is independent from the sponsor and competing interests; and reference to where further details about its charter can be found, if not in the protocol. Alternatively, an explanation of why a DMC is not needed	15
	21b	Description of any interim analyses and stopping guidelines, including who will have access to these interim results and make the final decision to terminate the trial	15
Harms	22	Plans for collecting, assessing, reporting, and managing solicited and spontaneously reported adverse events and other unintended effects of trial interventions or trial conduct	15
Auditing	23	Frequency and procedures for auditing trial conduct, if any, and whether the process will be independent from investigators and the sponsor	15
**Ethics and dissemination**			
Research ethics approval	24	Plans for seeking research ethics committee/institutional review board (REC/IRB) approval	15
Protocol amendments	25	Plans for communicating important protocol modifications (eg, changes to eligibility criteria, outcomes, analyses) to relevant parties (eg, investigators, REC/IRBs, trial participants, trial registries, journals, regulators)	15/16

For peer review only - http://bmjopen.bmj.com/site/about/guidelines.xhtml

Consent or assent	26a	Who will obtain informed consent or assent from potential trial participants or authorised surrogates, and how (see Item 32)	16
	26b	Additional consent provisions for collection and use of participant data and biological specimens in ancillary studies, if applicable	Na
Confidentiality	27	How personal information about potential and enrolled participants will be collected, shared, and maintained in order to protect confidentiality before, during, and after the trial	16
Declaration of interests	28	Financial and other competing interests for principal investigators for the overall trial and each study site	18/19
Access to data	29	Statement of who will have access to the final trial dataset, and disclosure of contractual agreements that limit such access for investigators	Na
Ancillary and post-trial care	30	Provisions, if any, for ancillary and post-trial care, and for compensation to those who suffer harm from trial participation	Na
Dissemination policy	31a	Plans for investigators and sponsor to communicate trial results to participants, healthcare professionals, the public, and other relevant groups (eg, via publication, reporting in results databases, or other data sharing arrangements), including any publication restrictions	16
	31b	Authorship eligibility guidelines and any intended use of professional writers	16
	31c	Plans, if any, for granting public access to the full protocol, participant-level dataset, and statistical code	Na
**Appendices**			
Informed consent materials	32	Model consent form and other related documentation given to participants and authorised surrogates	Supplementary
Biological specimens	33	Plans for collection, laboratory evaluation, and storage of biological specimens for genetic or molecular analysis in the current trial and for future use in ancillary studies, if applicable	Na

*It is strongly recommended that this checklist be read in conjunction with the SPIRIT 2013 Explanation & Elaboration for important clarification on the items. Amendments to the protocol should be tracked and dated. The SPIRIT checklist is copyrighted by the SPIRIT Group under the Creative Commons "Attribution-NonCommercial-NoDerivs 3.0 Unported" license.
